# Supplementary material for: Dissection of goadsporin biosynthesis by in vitro reconstitution leading to designer analogues expressed in vivo
Source: Nat Commun. 2017 Feb 6;8:14207. doi: 10.1038/ncomms14207 (PMC5303826; doi:10.1038/ncomms14207)
Supplement: Supplementary Information — Supplementary Figures, Supplementary Tables and Supplementary References. [file ncomms14207-s1.pdf]

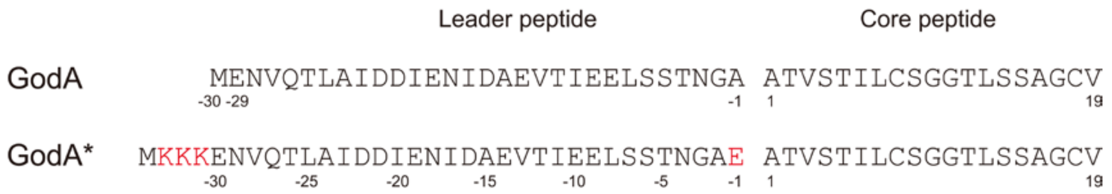

Supplementary Figure 1. Amino acid sequences of GodA and GodA*. Inserted residues are colored red. Numbers indicate the position of each residue.

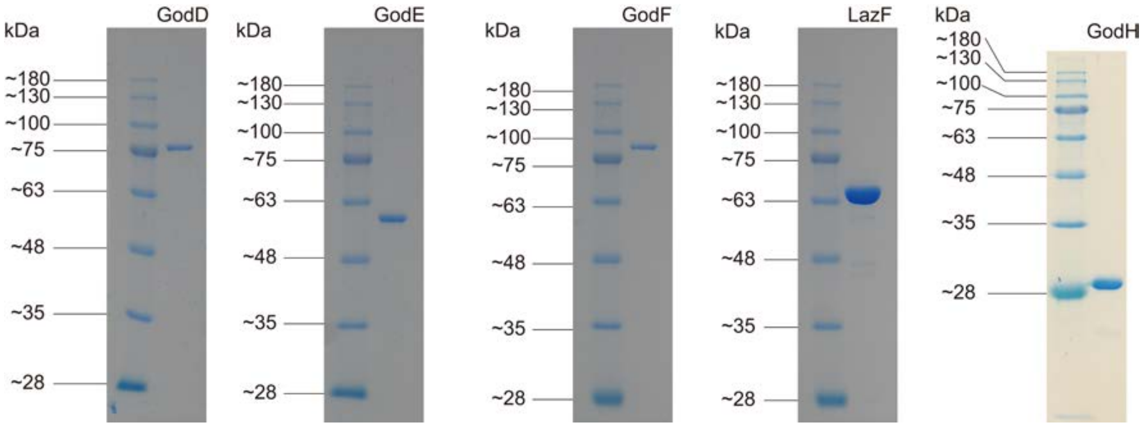

Supplementary Figure 2. SDS-PAGE analysis of purified recombinant proteins. 12.5% acrylamide gel was used for GodD, GodE, GodF, and LazF. 15% acrylamide gel was used for GodH. BLUE Star Prestained Protein-Ladder (NIPPON Genetics Co, Ltd.) was used as a molecular weight marker.

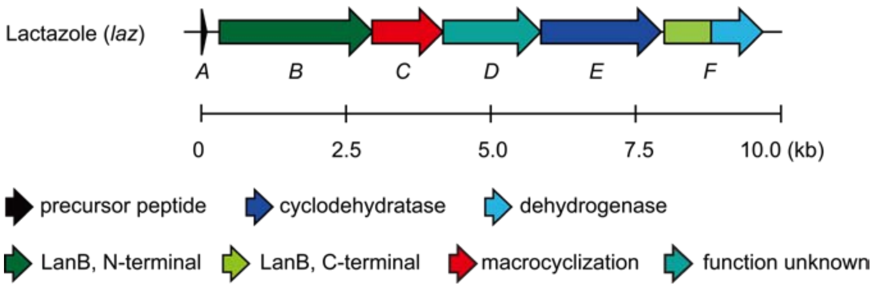

Supplementary Figure 3. The biosynthetic gene cluster for lactazoles.

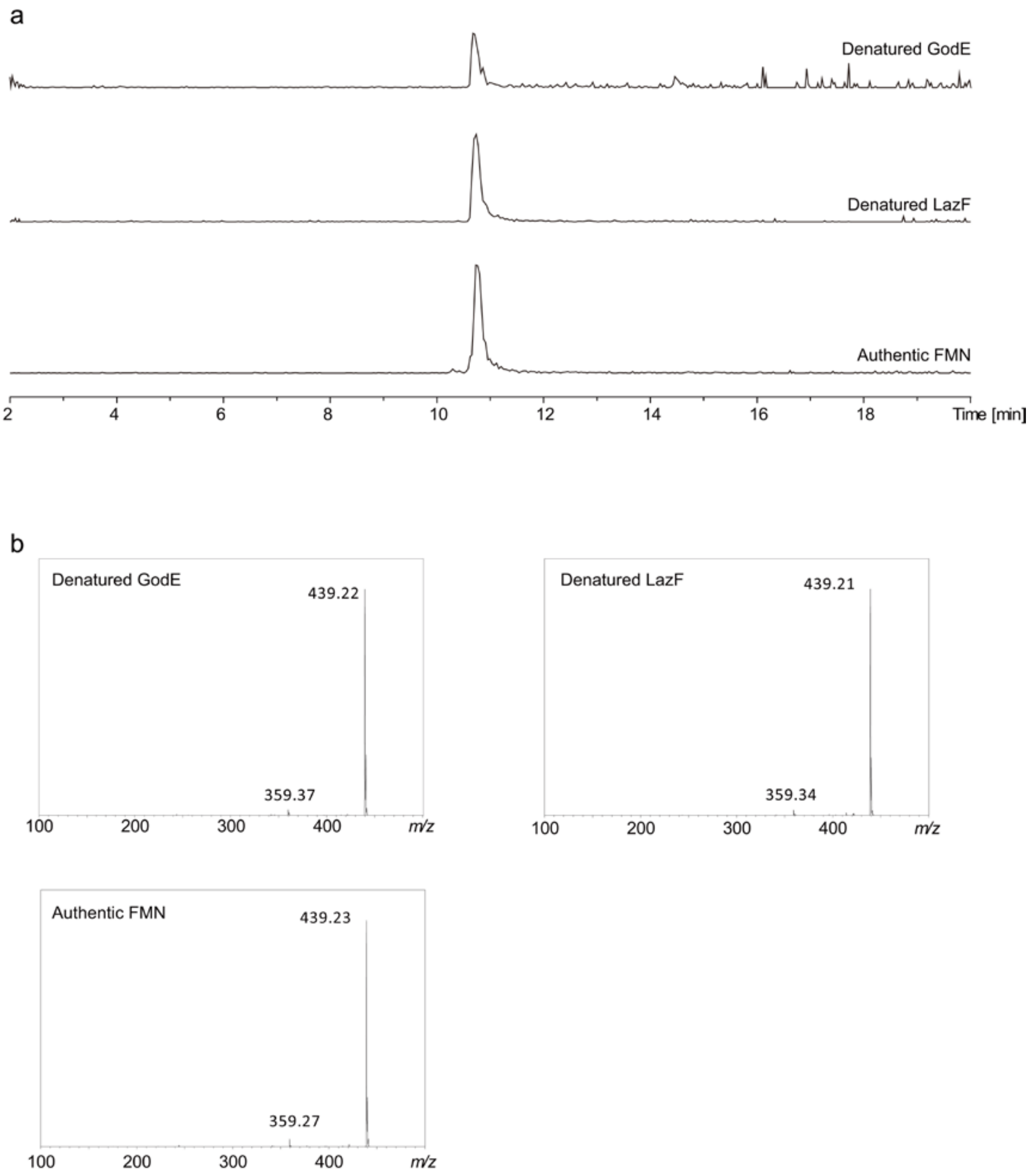

Supplementary Figure 4. LC-MS analysis of flavin co-factor bound to recombinant GodE and LazF. a, chromatograms extracted at *m/z* 457 $[M+H]^+$ were shown. Chromatograms from denatured GodE and LazF were compared with that of authentic flavin mononucleotide (FMN). **b**, MS^2 spectra of FMN (*m/z* 457) detected in each sample.

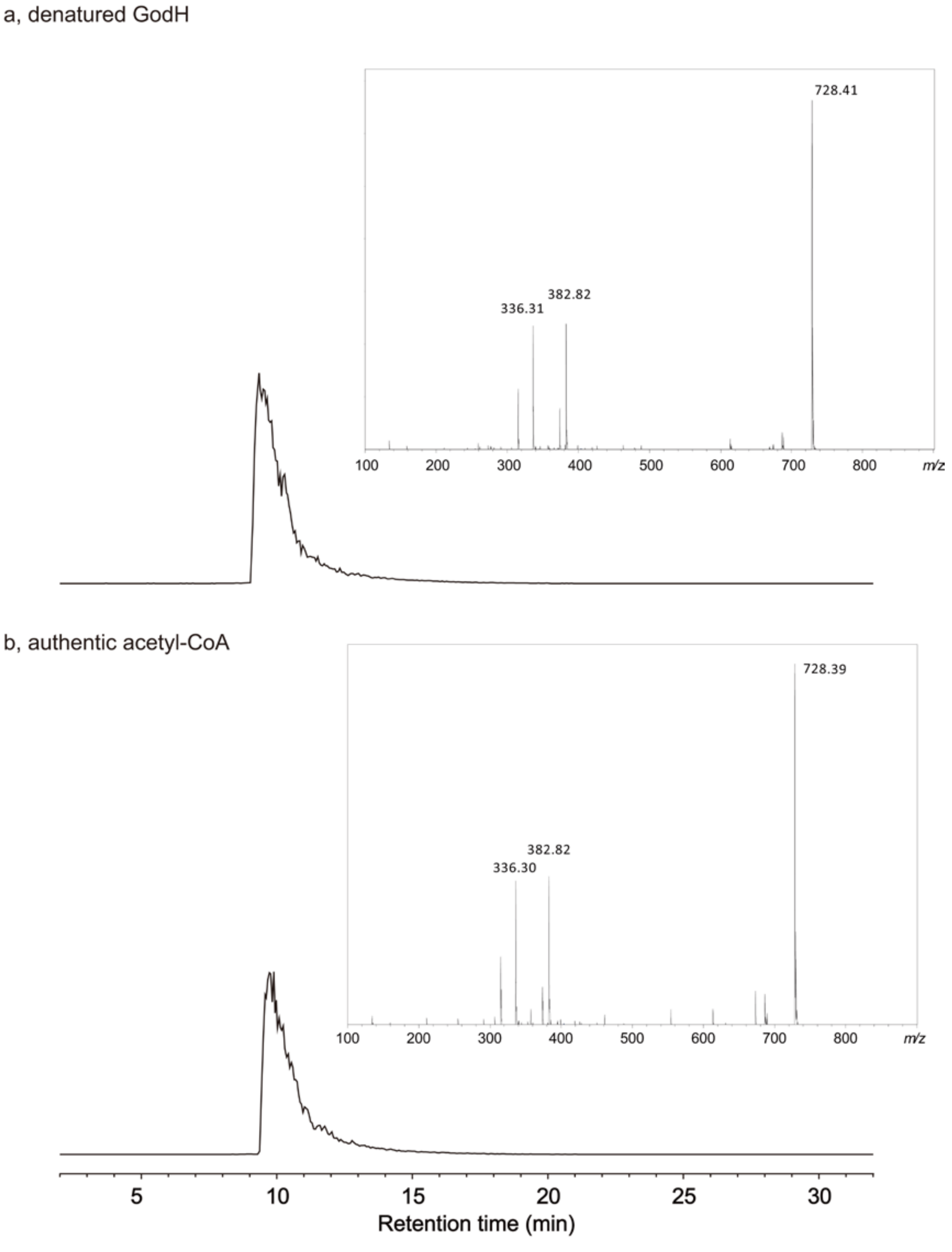

Supplementary Figure 5. LC-MS analysis of co-factor bound to recombinant GodH.

The methanol extract of GodH (**a**) was compared with authentic acetyl-CoA (**b**), Chromatograms were extracted at *m/z* 808 $[M-H]^-$. MS^2 spectra derived from *m/z* 808

precursor ions were shown in boxes.

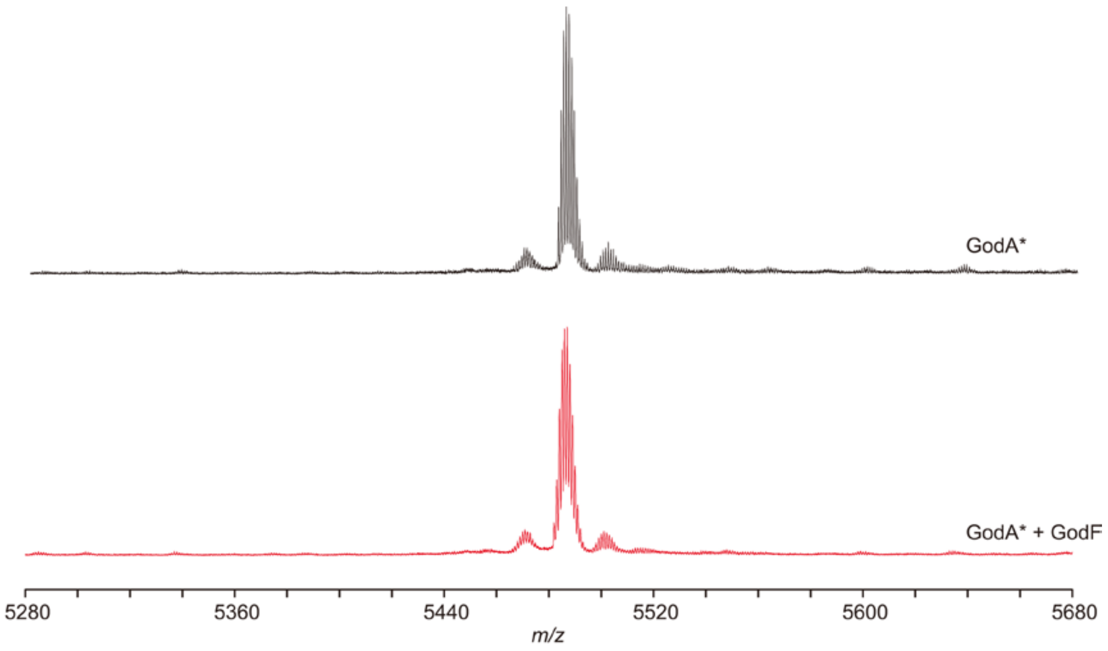

Supplementary Figure 6. MALDI-TOF-MS analysis of the reaction product of GodF reaction with GodA*. Red line is from enzymatic reaction and black line from control. Calculated and observed *m/z* values in MALDI-TOF-MS analyses are summarized in Supplementary Data.

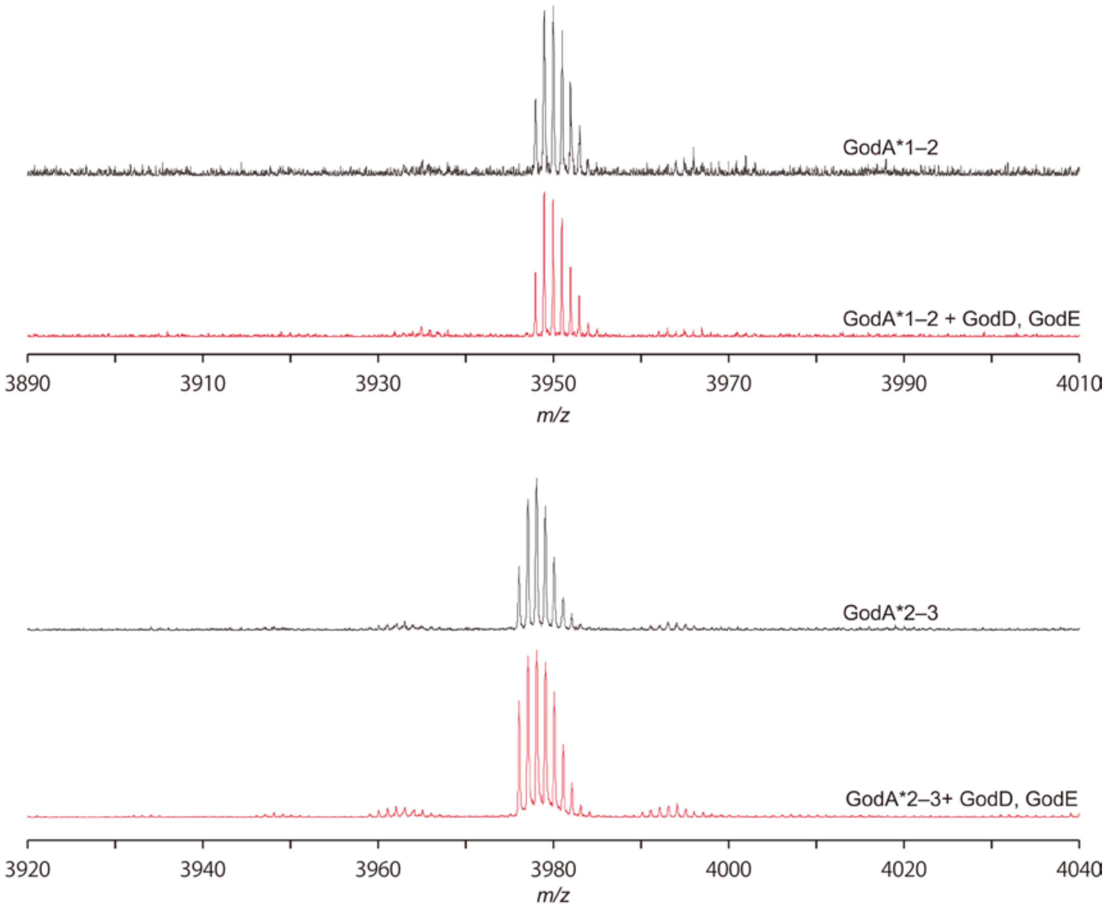

Supplementary Figure 7. MALDI-TOF-MS analysis of the reaction catalyzed by GodD and GodE with GodA*1–2 and GodA*2–3. Red lines are from enzymatic reactions and black lines from control. Calculated and observed *m/z* values in MALDI-TOF-MS analyses are summarized in Supplementary Data.

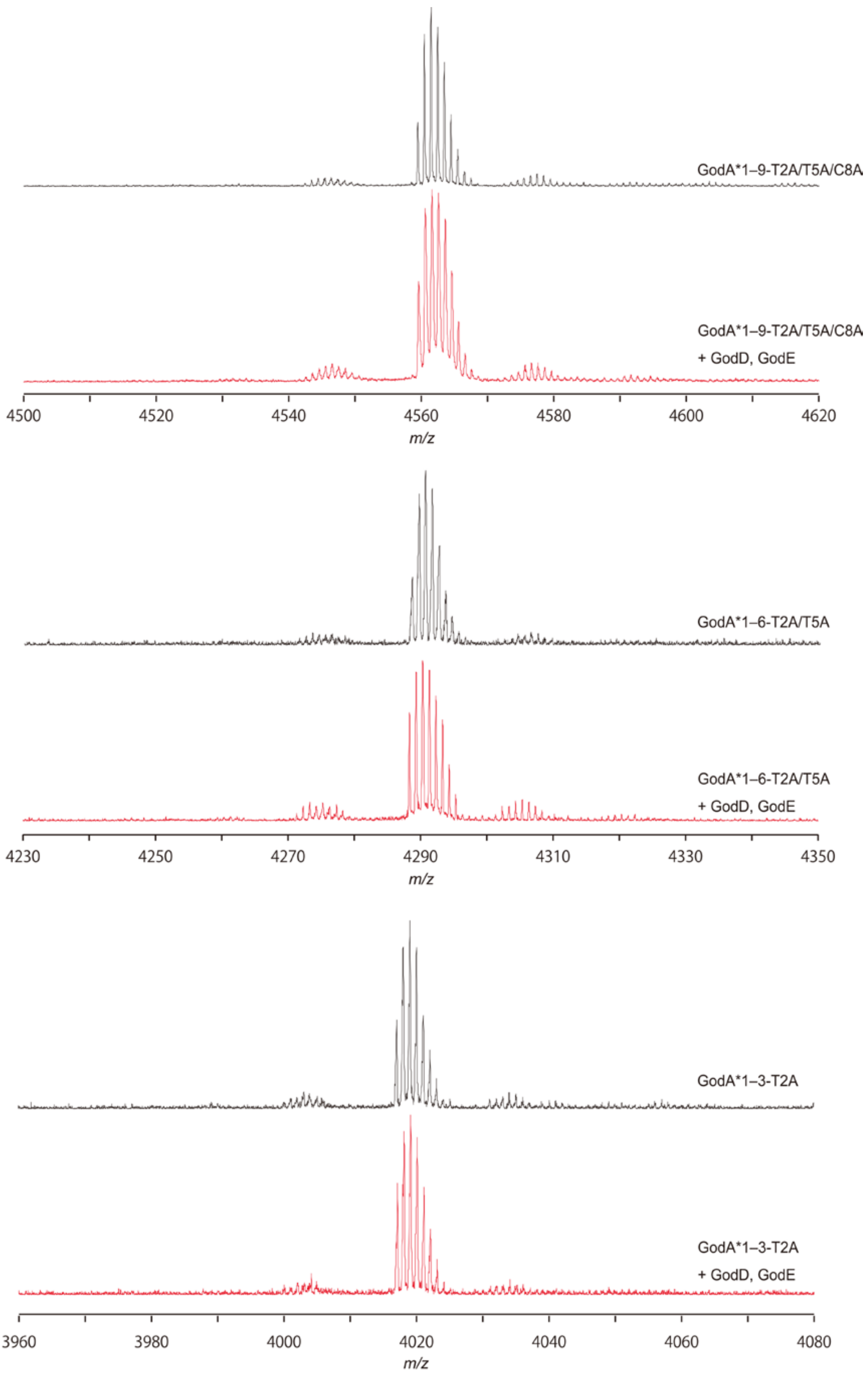
GodA*1–9-T2A/T5A/C8A
GodA*1–9-T2A/T5A/C8A
+ GodD, GodE
4500
4520
4540
4560
4580
4600
4620
m/z
GodA*1–6-T2A/T5A
GodA*1–6-T2A/T5A
+ GodD, GodE
4230
4250
4270
4290
4310
4330
4350
m/z
GodA*1–3-T2A
GodA*1–3-T2A
+ GodD, GodE
3960
3980
4000
4020
4040
4060
4080
m/z

Supplementary Figure 8. MALDI-TOF-MS analysis of the reaction catalyzed by GodD and GodE with GodA*1–9-T2A/T5A/C8A, GodA*1–6-T2A/T5A, and GodA*1–3-T2A. Red lines are from enzymatic reactions and black lines from control. Calculated and observed *m/z* values in MALDI-TOF-MS analyses are summarized in Supplementary Data.

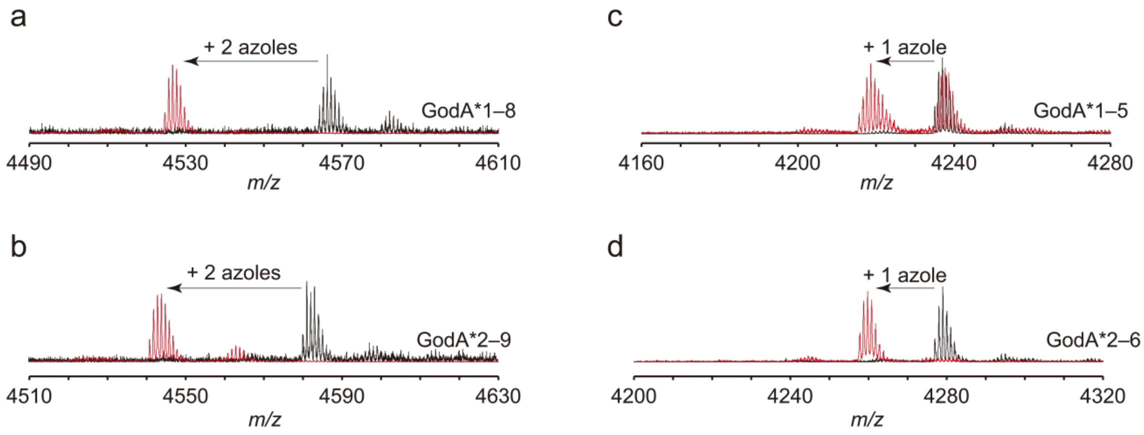

Supplementary Figure 9. MALDI-TOF-MS analysis of the reaction catalyzed by GodD and GodE with truncated mutants. GodA*1–8 (**a**), GodA*2–9 (**b**), GodA*1–5 (**c**), and GodA*2–6 (**d**) were used as substrates. Red lines are from enzymatic reactions and black lines from control. Calculated and observed *m/z* values in MALDI-TOF-MS analyses are summarized in Supplementary Data.

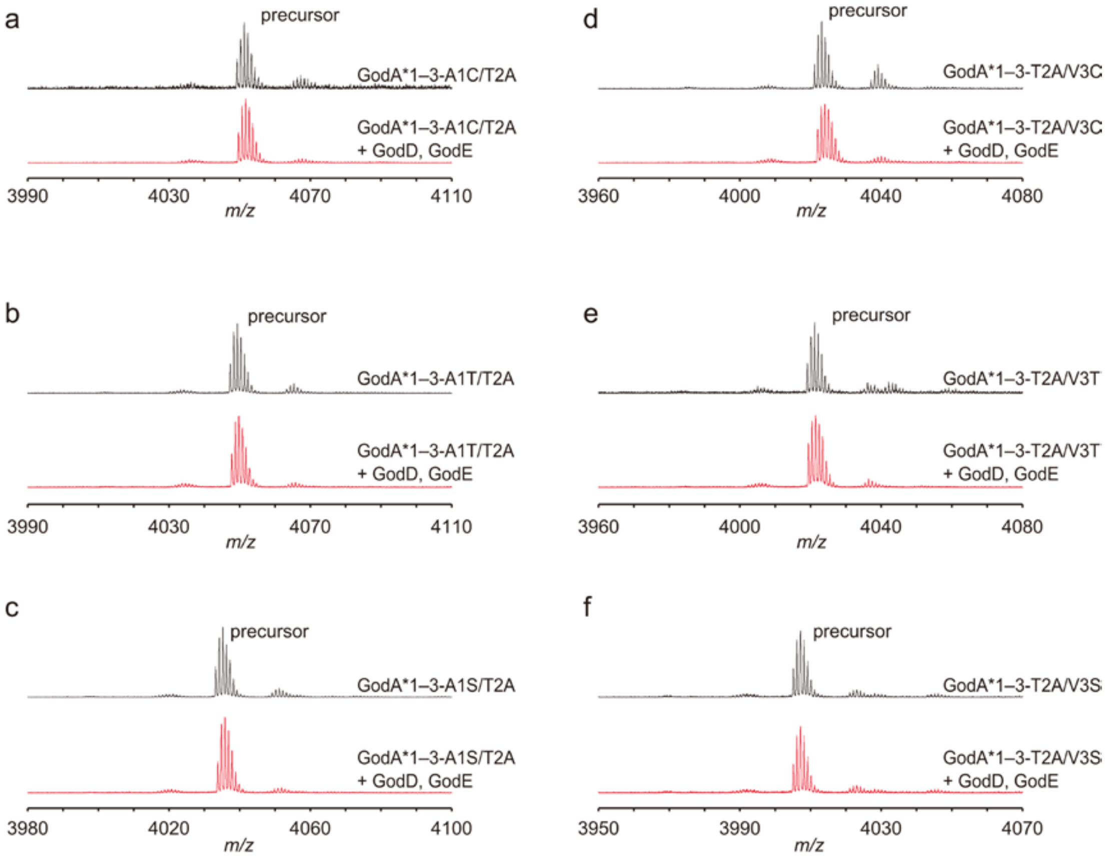

Supplementary Figure 10. MALDI-TOF-MS analysis of the reaction catalyzed by GodD and GodE with GodA*1–-3 mutants whose X_1/X_2 residues were substituted with S/T/C residues. GodA*1–3-A1C/T2A (**a**), GodA*1–3-A1T/T2A (**b**), GodA*1–3-A1S/T2A (**c**), GodA*1–3-T2A/V3C (**d**), GodA*1–3-T2A/V3T (**e**), and GodA*1–3-T2A/V3S (**f**) were used as substrates. Red lines are from enzymatic reactions and black lines from control. Calculated and observed *m/z* values in MALDI-TOF-MS analyses are summarized in Supplementary Data.

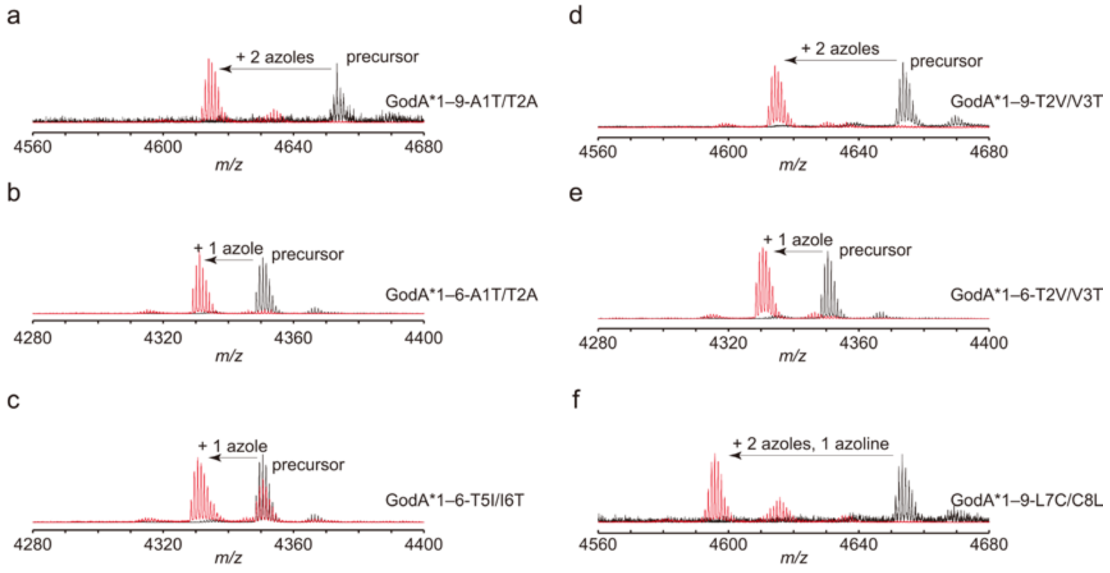

Supplementary Figure 11. MALDI-TOF-MS analysis of the reaction catalyzed by GodD and GodE with GodA*1–9-A1T/T2A (a), GodA*1–6-A1T/T2A (b), GodA*1–6-T5I/I6T (c), GodA*1–9-T2V/V3T (d), GodA*1–6-T2V/V3T (e), and GodA*1–9-L7C/C8L (f). Red lines are from enzymatic reactions and black lines from control. Calculated and observed *m/z* values in MALDI-TOF-MS analyses are summarized in Supplementary Data.

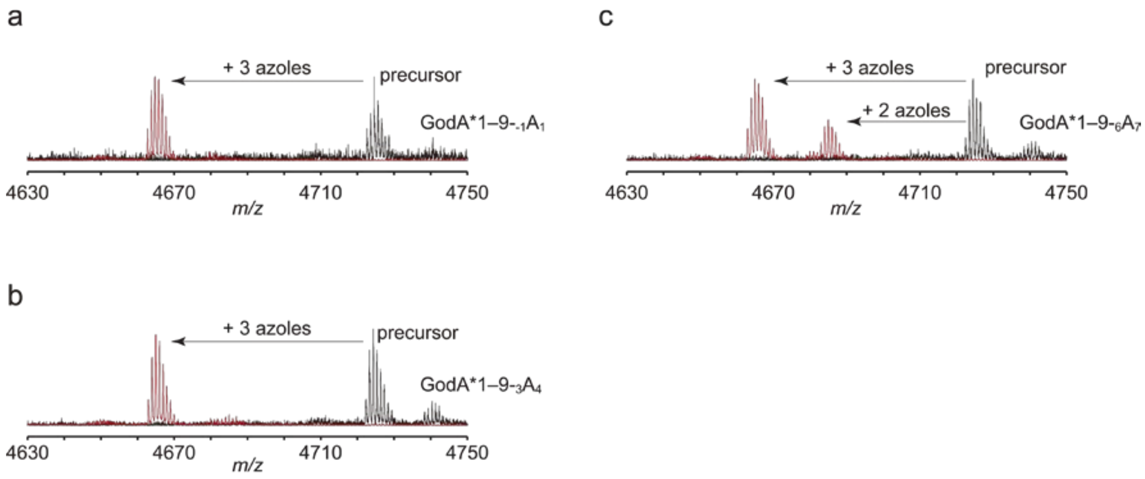

Supplementary Figure 12. MALDI-TOF-MS analysis of the reaction catalyzed by GodD and GodE with GodA*1–9-$_{-1}$A$_1$ (a), GodA*1–9-$_3$A$_4$ (b), and GodA*1–9-$_6$A$_7$ (c). Red lines are from enzymatic reactions and black lines from control. Calculated and observed *m/z* values in MALDI-TOF-MS analyses are summarized in Supplementary Data.

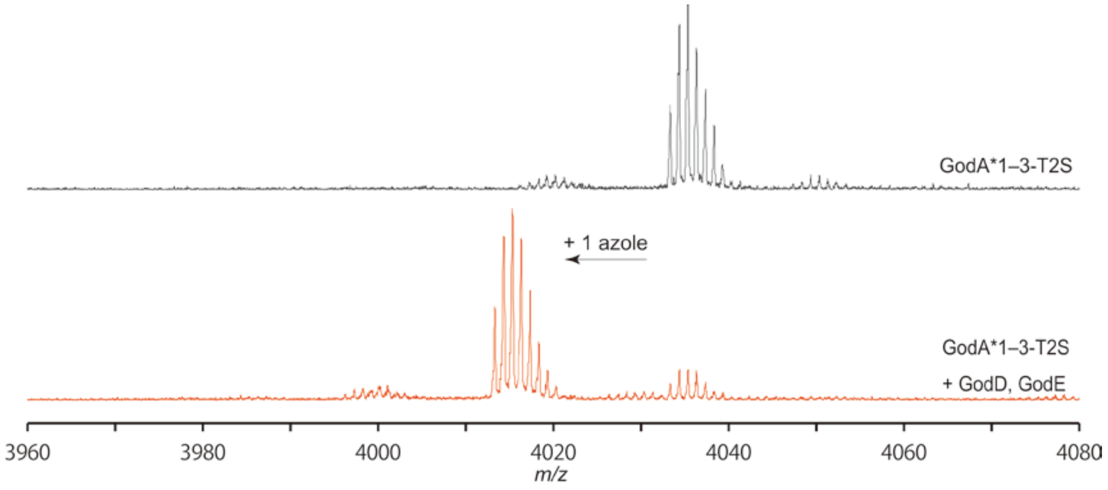

Supplementary Figure 13. MALDI-TOF-MS analysis of the reaction catalyzed by GodD and GodE with GodA*1–3-T2S. Red line is from enzymatic reaction and black line from control. Calculated and observed *m/z* values in MALDI-TOF-MS analyses are summarized in Supplementary Data.

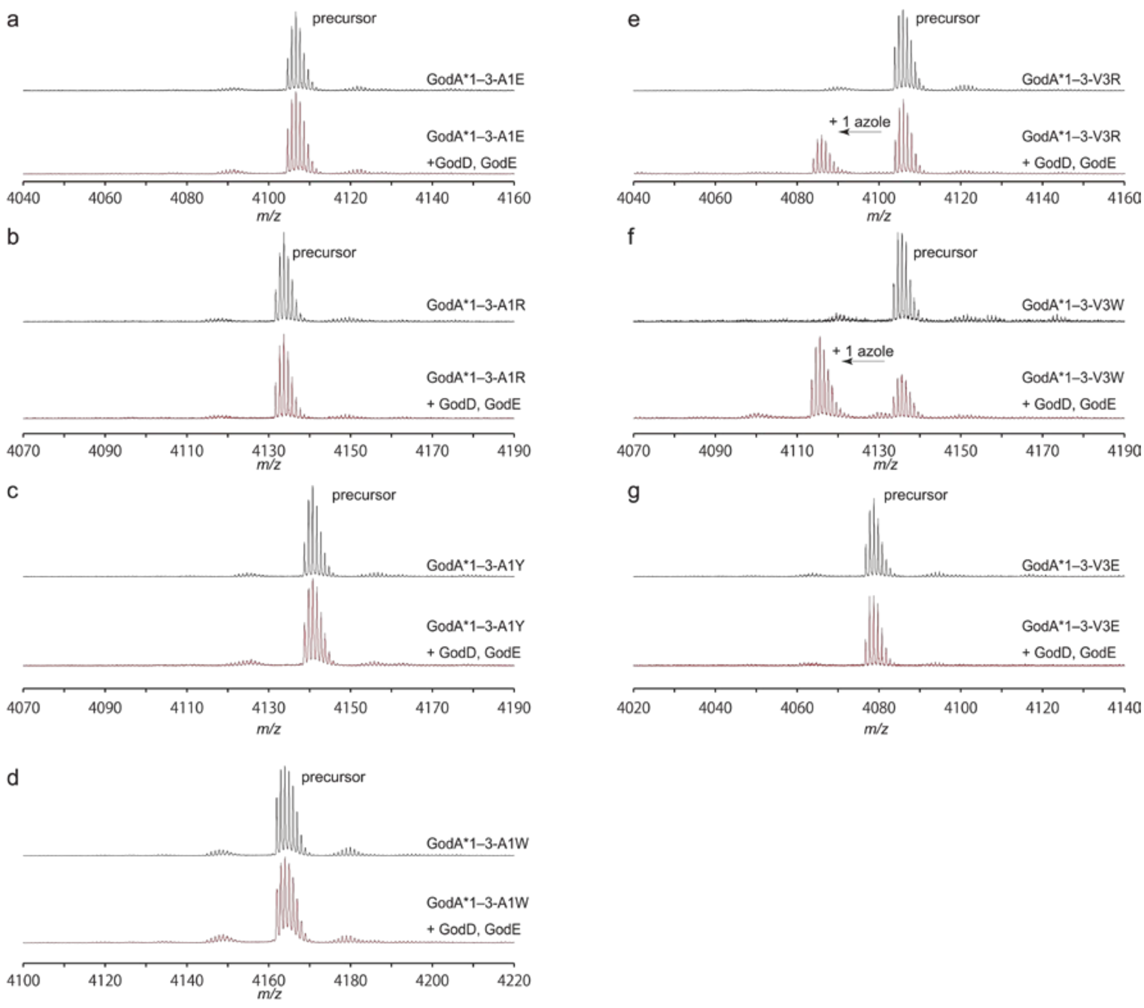

Supplementary Figure 14. MALDI-TOF-MS analysis of the reaction catalyzed by GodD and GodE with GodA*1–3 mutants which harbor the mutation at X_1/X_2 residues. GodA*1–3-A1E (**a**), GodA*1–3-A1R (**b**), GodA*1–3-A1Y (**c**), GodA*1–3-A1W (**d**), GodA*1–3-V3R (**e**), GodA*1–3-V3W (**f**), and GodA*1–3-V3E (**g**) were used as substrates. Red lines are from enzymatic reactions and black lines from control. Calculated and observed *m/z* values in MALDI-TOF-MS analyses are summarized in Supplementary Data.

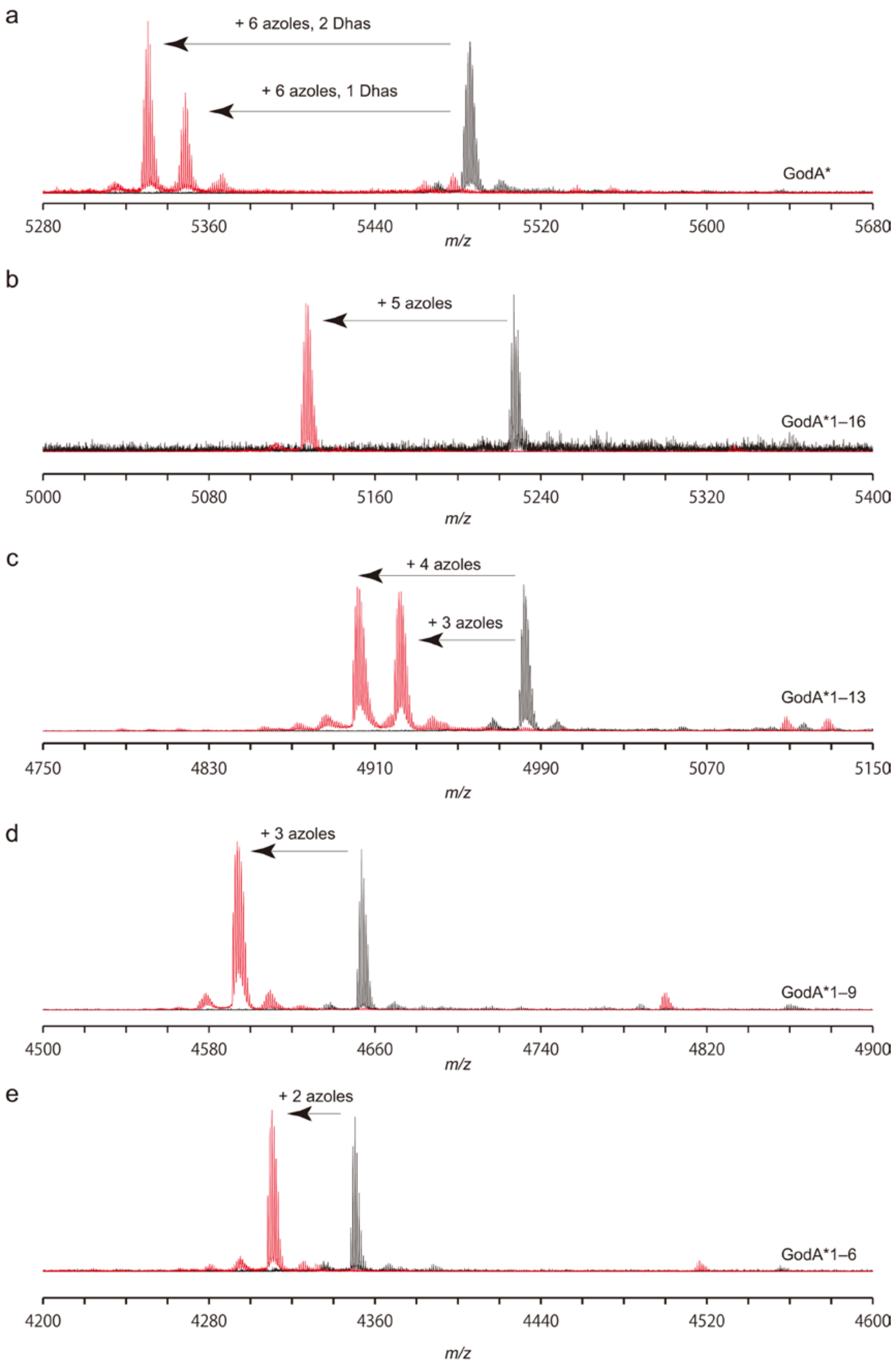

a
+ 6 azoles, 2 Dhas
+ 6 azoles, 1 Dhas
GodA*
5280
5360
5440
5520
5600
5680
m/z
b
+ 5 azoles
GodA*1–16
5000
5080
5160
5240
5320
5400
m/z
c
+ 4 azoles
+ 3 azoles
GodA*1–13
4750
4830
4910
4990
5070
5150
m/z
d
+ 3 azoles
GodA*1–9
4500
4580
4660
4740
4820
4900
m/z
e
+ 2 azoles
GodA*1–6
4200
4280
4360
4440
4520
4600
m/z

Supplementary Figure 15. MALDI-TOF-MS analysis of the reaction catalyzed by GodD, GodE, GodF, and LazF with GodA* (a), GodA*1–16 (b), GodA*1–13 (c), GodA*1-–9 (d) and GodA*1–6 (e). Red lines are from enzymatic reactions and black lines from control. Calculated and observed *m/z* values in MALDI-TOF-MS analyses are summarized in Supplementary Data.

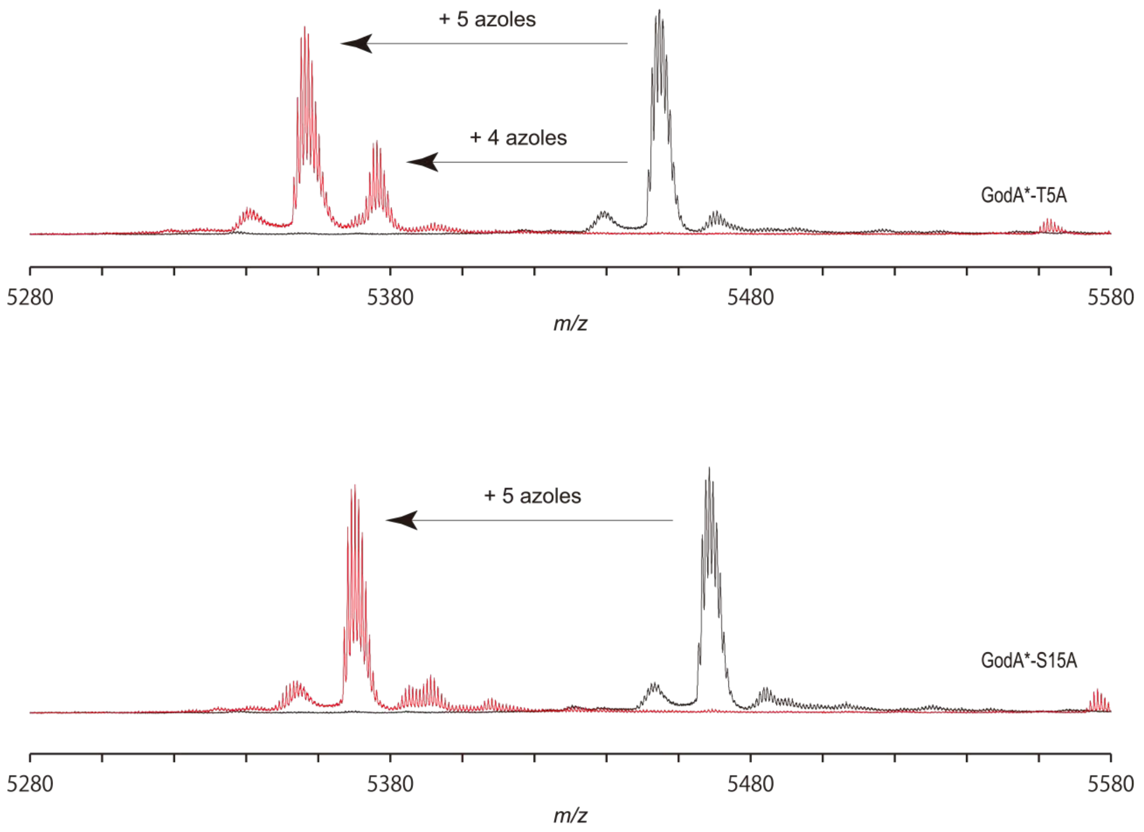

Supplementary Figure 16. MALDI-TOF-MS analysis of the reaction catalyzed by GodD, GodE, GodF, and LazF with GodA*-T5A and GodA*-S15A. Red lines are from enzymatic reactions and black lines from control. Calculated and observed *m/z* values in MALDI-TOF-MS analyses are summarized in Supplementary Data.

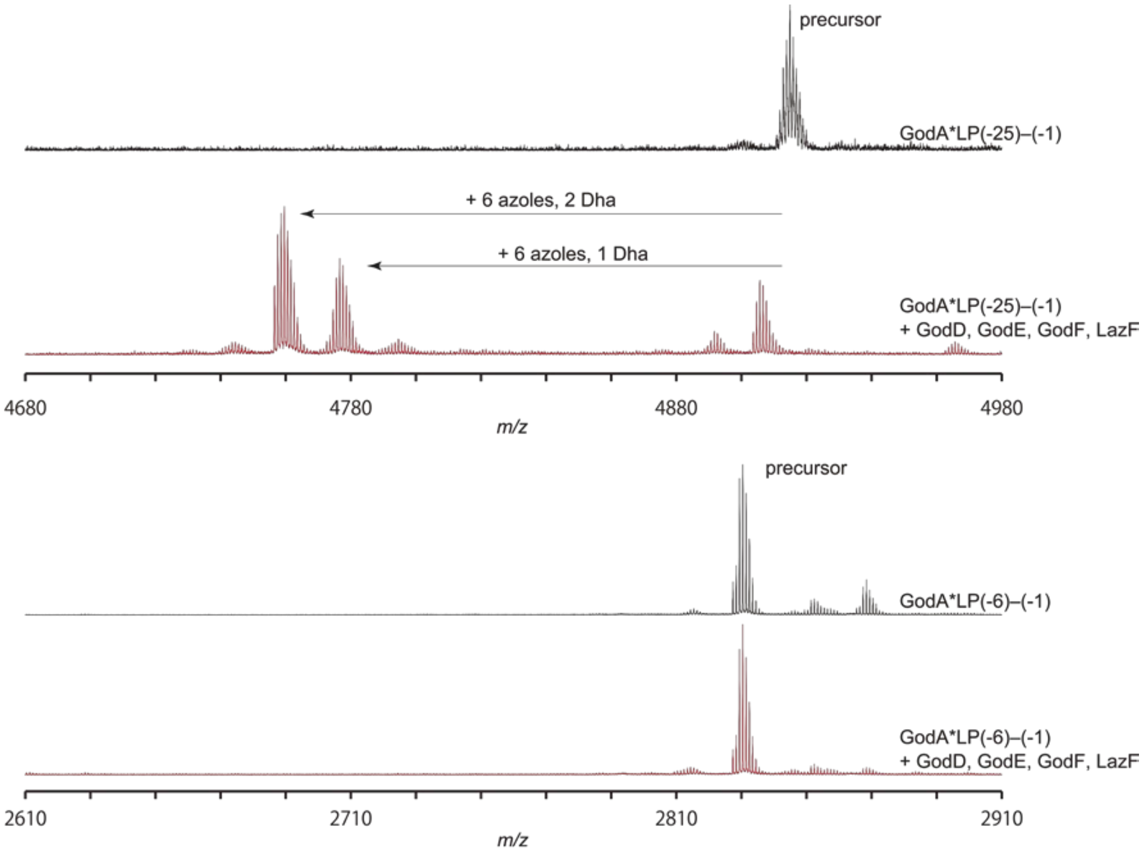

Supplementary Figure 17. MALDI-TOF-MS analysis of the reaction catalyzed by GodD, GodE, GodF, and LazF with GodA*LP(-25)–(-1) and GodA*LP(-6)–(-1) Red lines are from enzymatic reactions and black lines from control. Calculated and observed *m/z* values in MALDI-TOF-MS analyses are summarized in Supplementary Data.

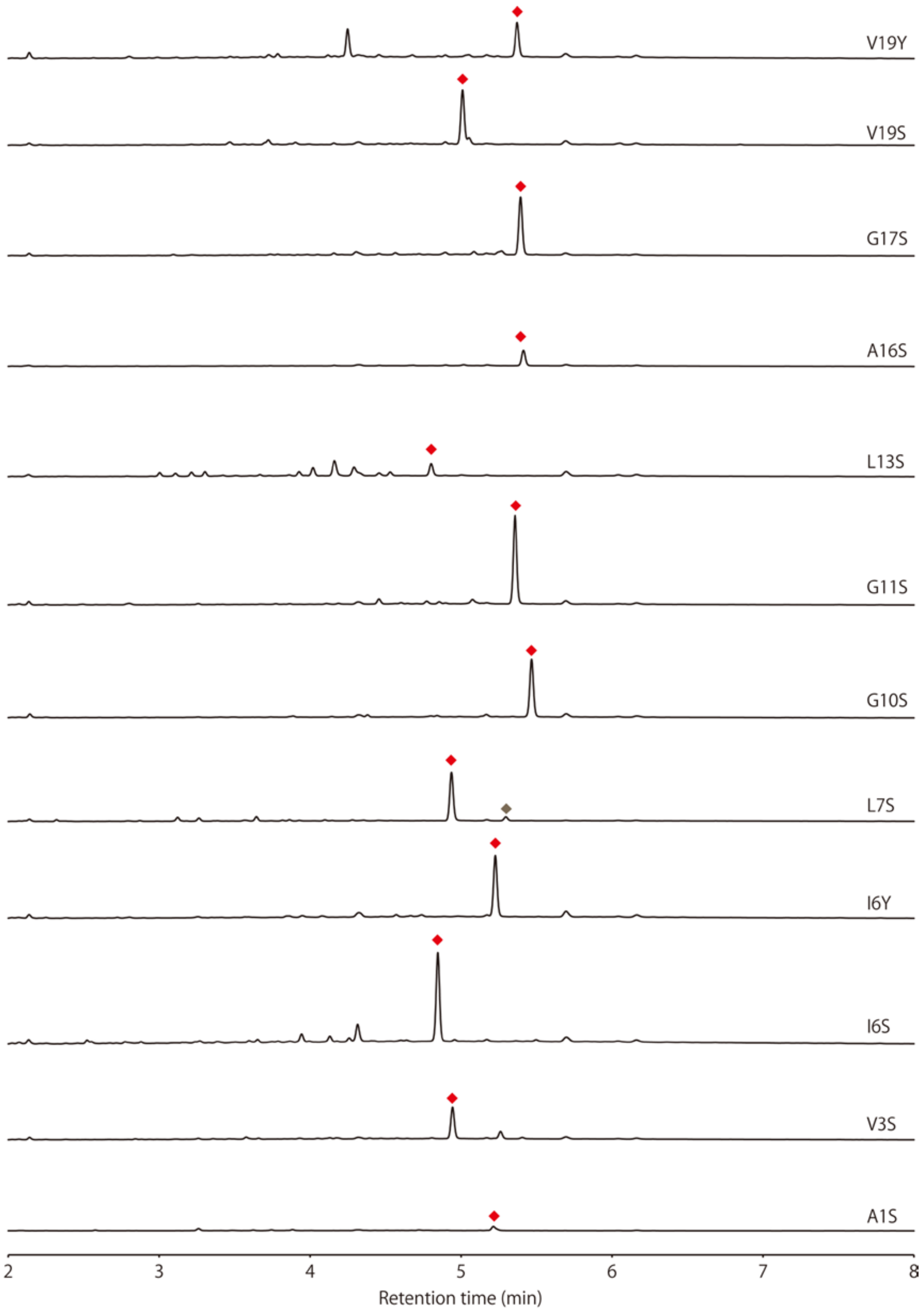

Supplementary Figure 18. LC-MS analyses of the metabolites produced by *Streptomyces* sp. TP-A0584 *ΔgodA* harboring pGODR and *godA* mutants. Red

diamondss indicate the major GS derivatives. Gray diamond indicates the minor derivative mentioned in the text. Chromatograms were extracted at 254 nm.

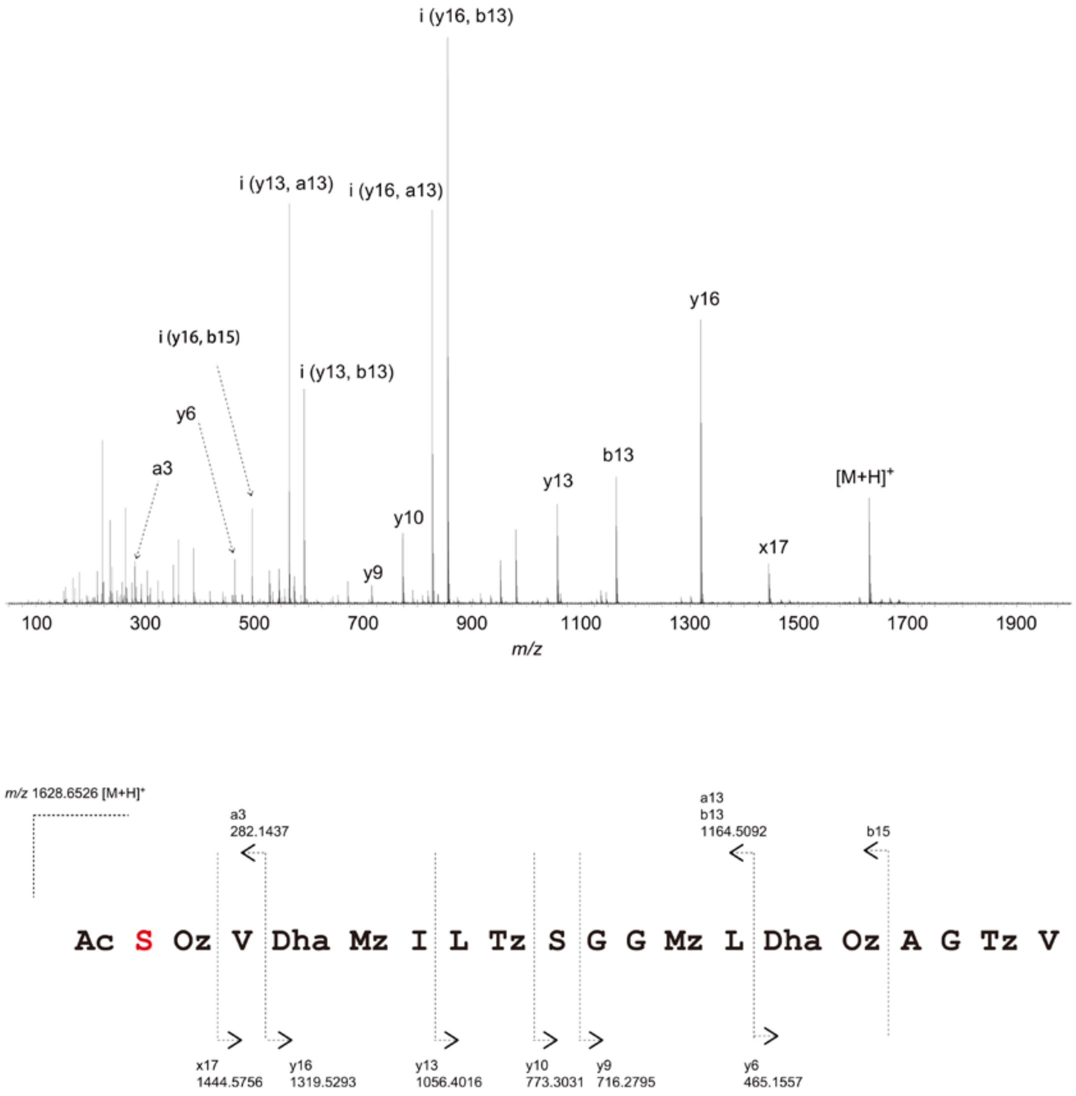

Supplementary Figure 19. MS fragmentation analysis of A1S. MS^E spectrum and assignment for fragmentation are shown. The labels, i (ym, a/bn) indicate the internal peptide fragment derived from fragments ym and a/bn. The fragment, i (y16, b15) was observed as a divalent ion.

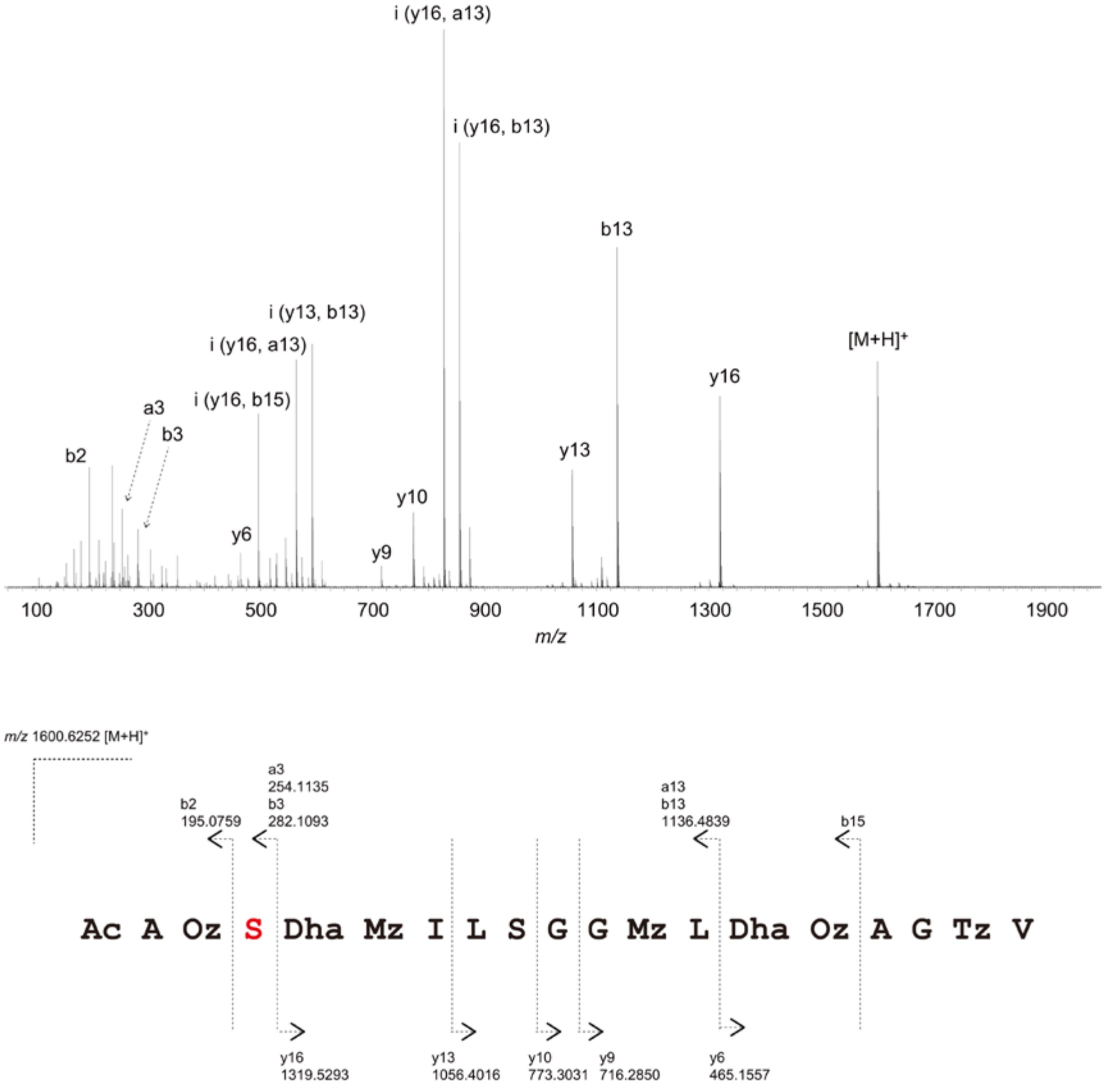

Supplementary Figure 20. MS fragmentation analysis of V3S. MS^E spectrum and assignment for fragmentation are shown. The labels, i (ym, a/bn) indicat the internal peptide fragment derived from fragments ym and a/bn. The fragment, i (y16, b15) was observed as a divalent ion.

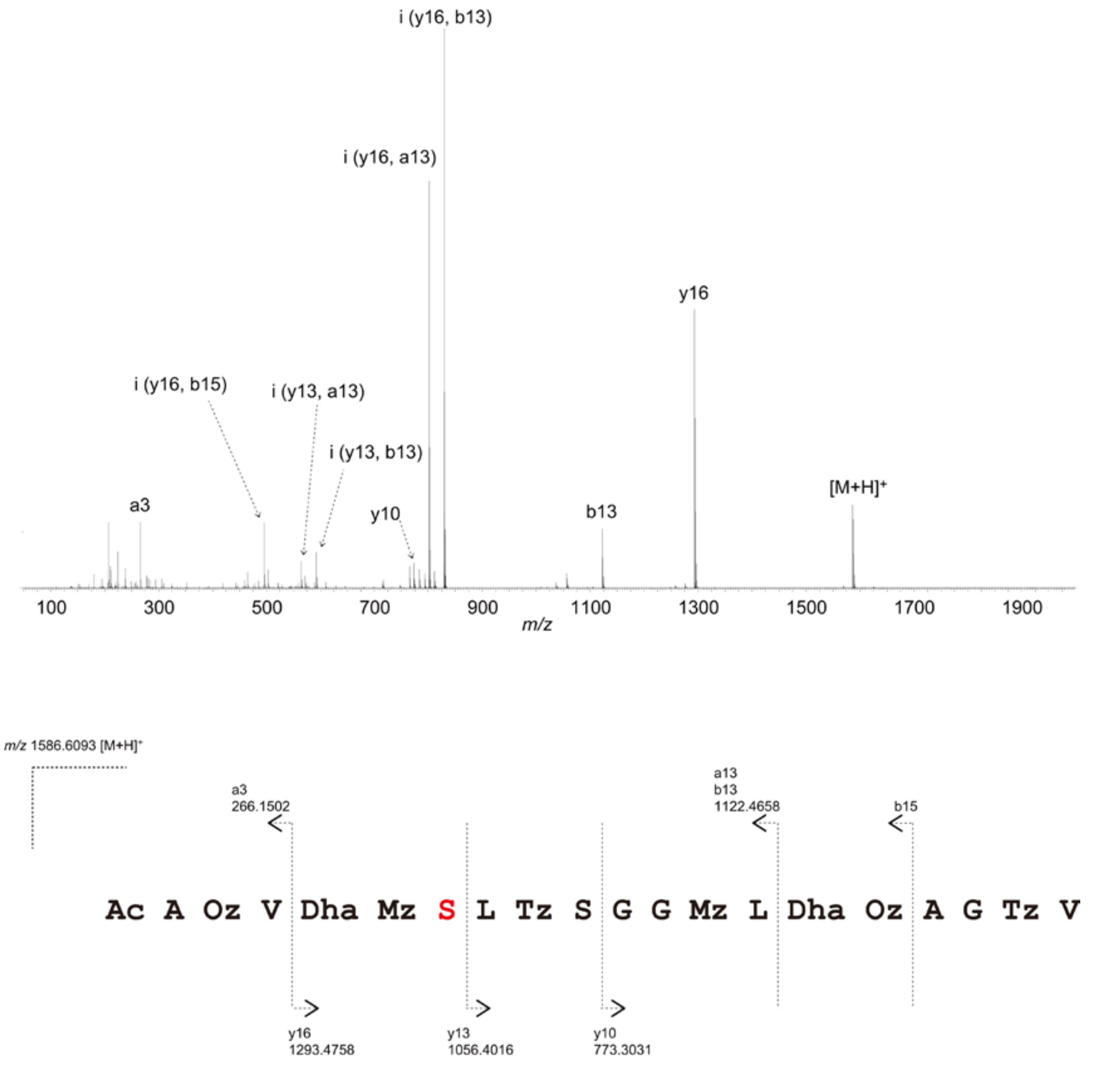

Supplementary Figure 21. MS fragmentation analysis of I6S. MS^E spectrum and assignment for fragmentation are shown. The labels, i (ym, a/bn) indicate the internal peptide fragment derived from fragments ym and a/bn. The fragment, i (y16, b15) was observed as a divalent ion.

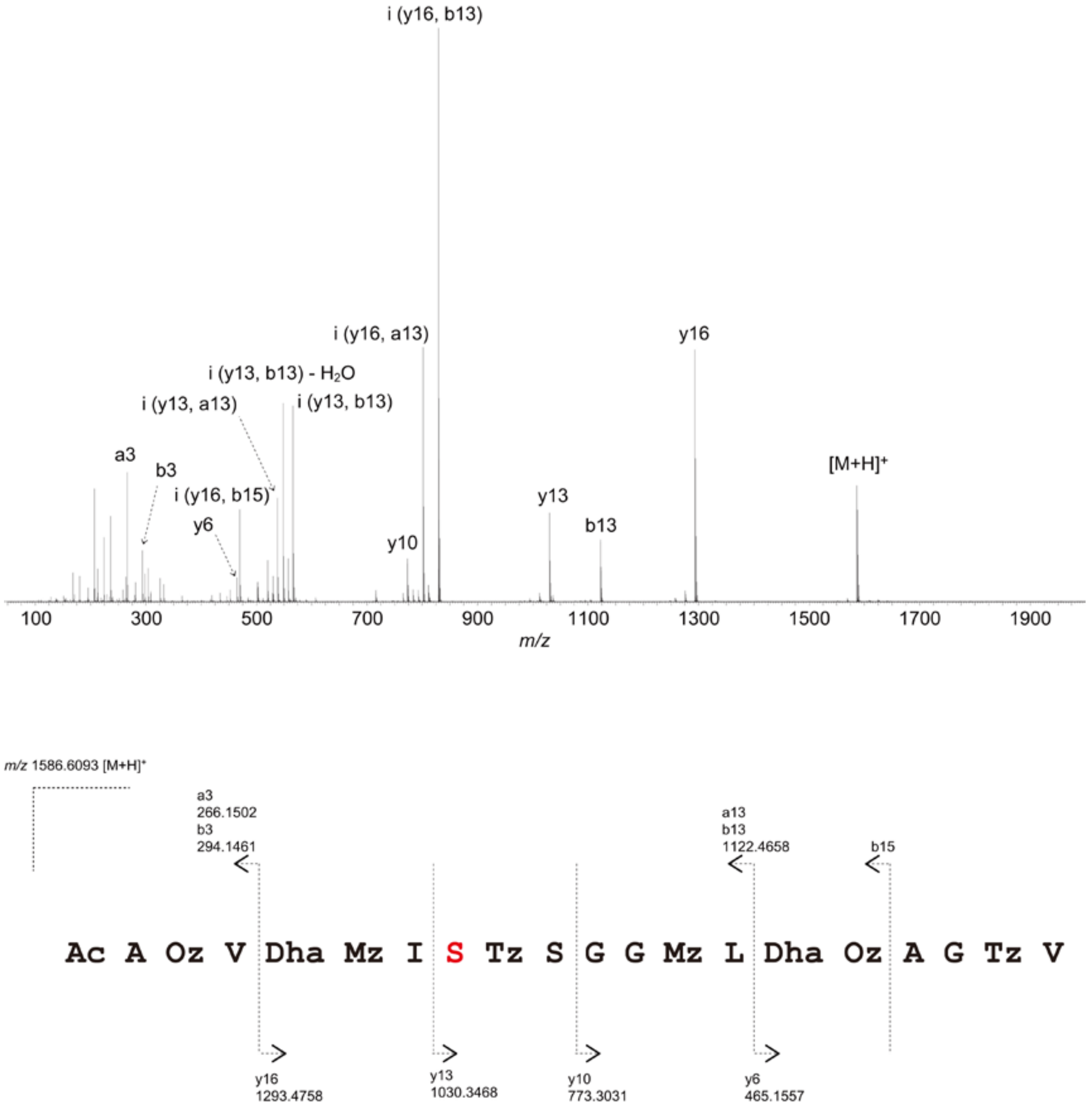

Supplementary Figure 22. MS fragmentation analysis of the major product of L7S.

MS^{E} spectrum and assignment for fragmentation are shown. The labels, i (ym, a/bn) indicate the internal peptide fragment derived from fragments ym and a/bn. The fragment, i (y16, b15) was observed as a divalent ion.

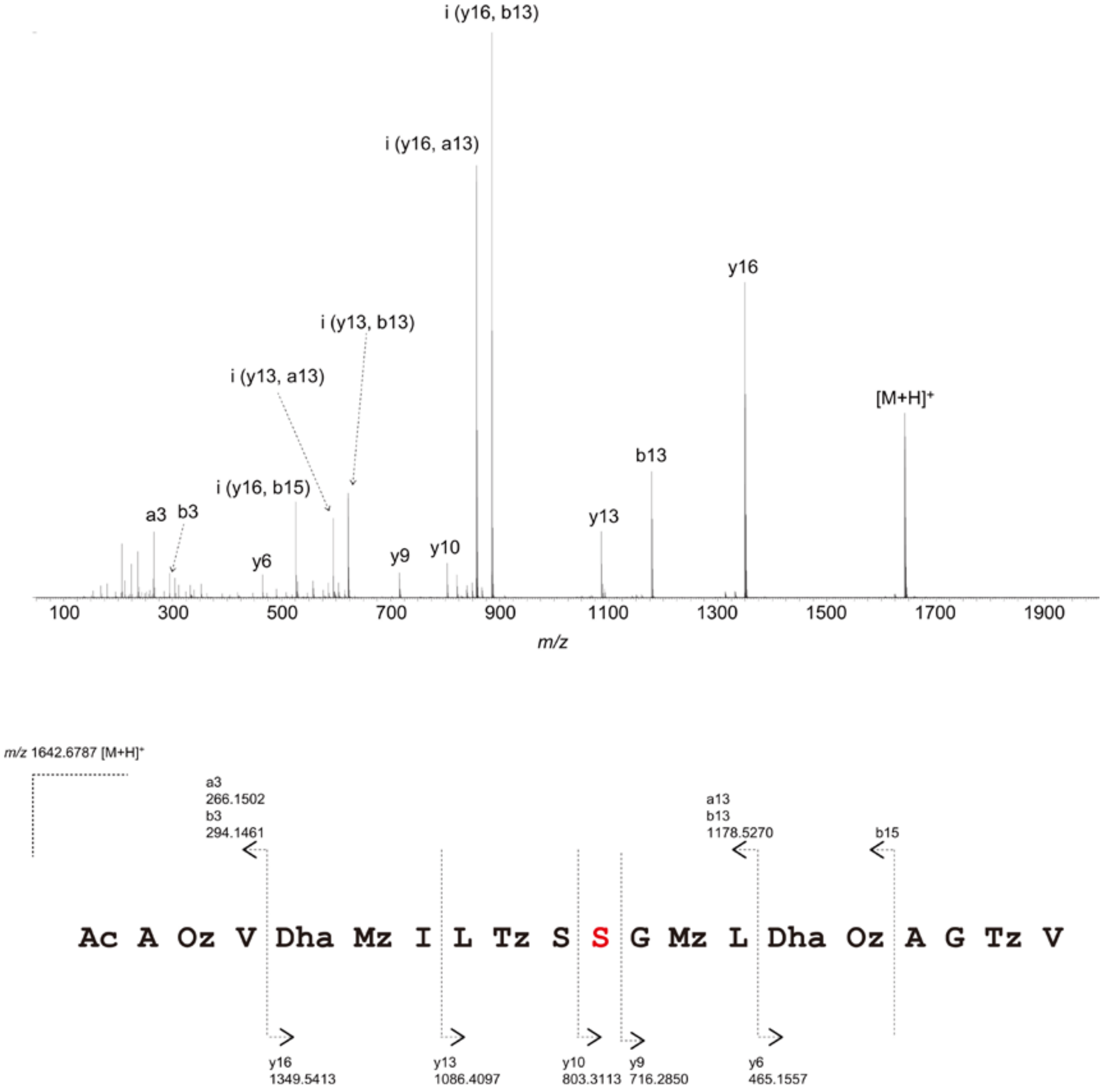

Supplementary Figure 23. MS fragmentation analysis of G10S. MS^E spectrum and assignment for fragmentation are shown. The labels, i (ym, a/bn) indicate the internal peptide fragment derived from fragments ym and a/bn. The fragment, i (y16, b15) was observed as a divalent ion.

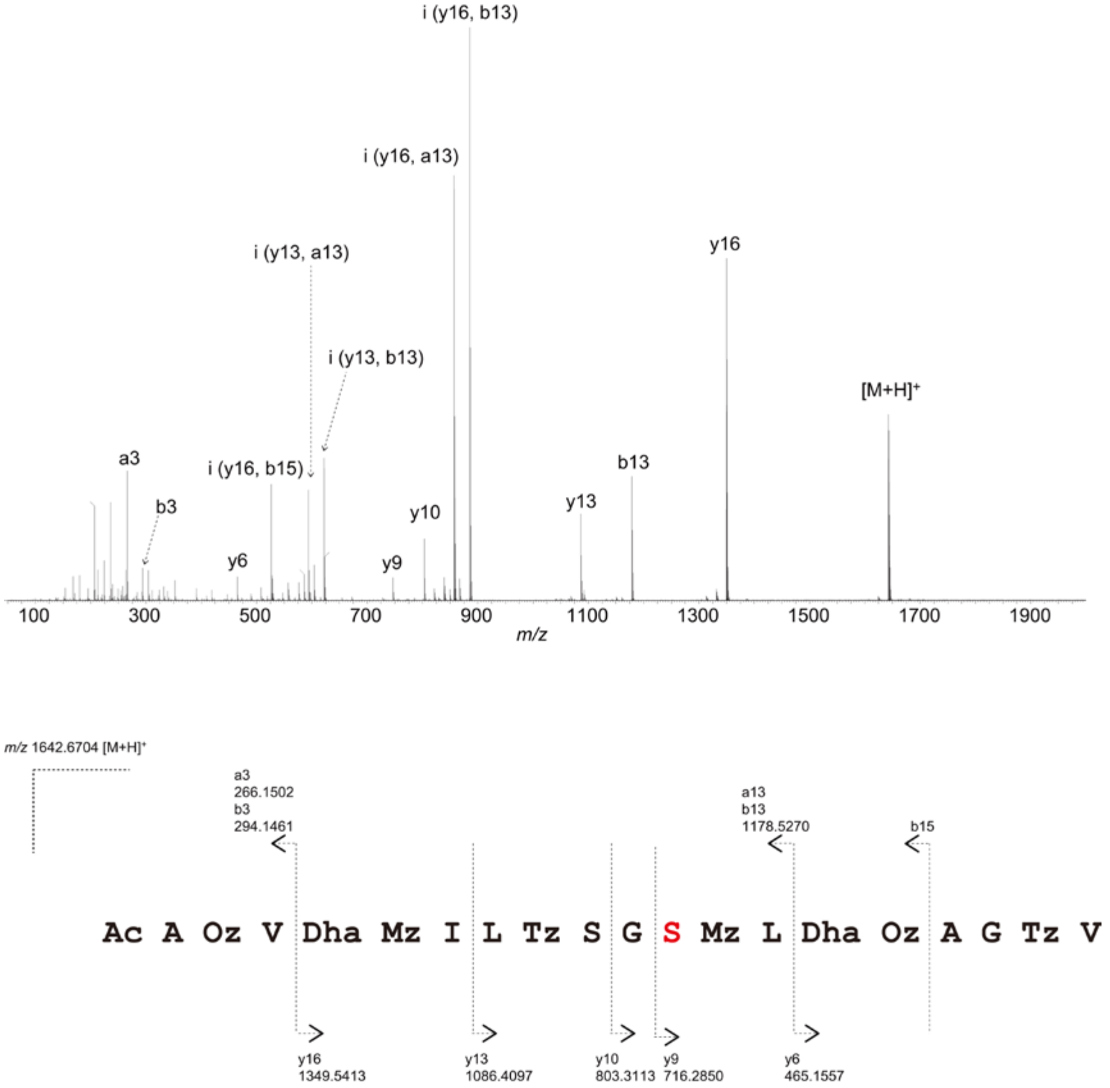

Supplementary Figure 24. MS fragmentation analysis of G11S. MS^E spectrum and assignment for fragmentation are shown. The labels, i (ym, a/bn) indicate the internal peptide fragment derived from fragments ym and a/bn. The fragment, i (y16, b15) was observed as a divalent ion.

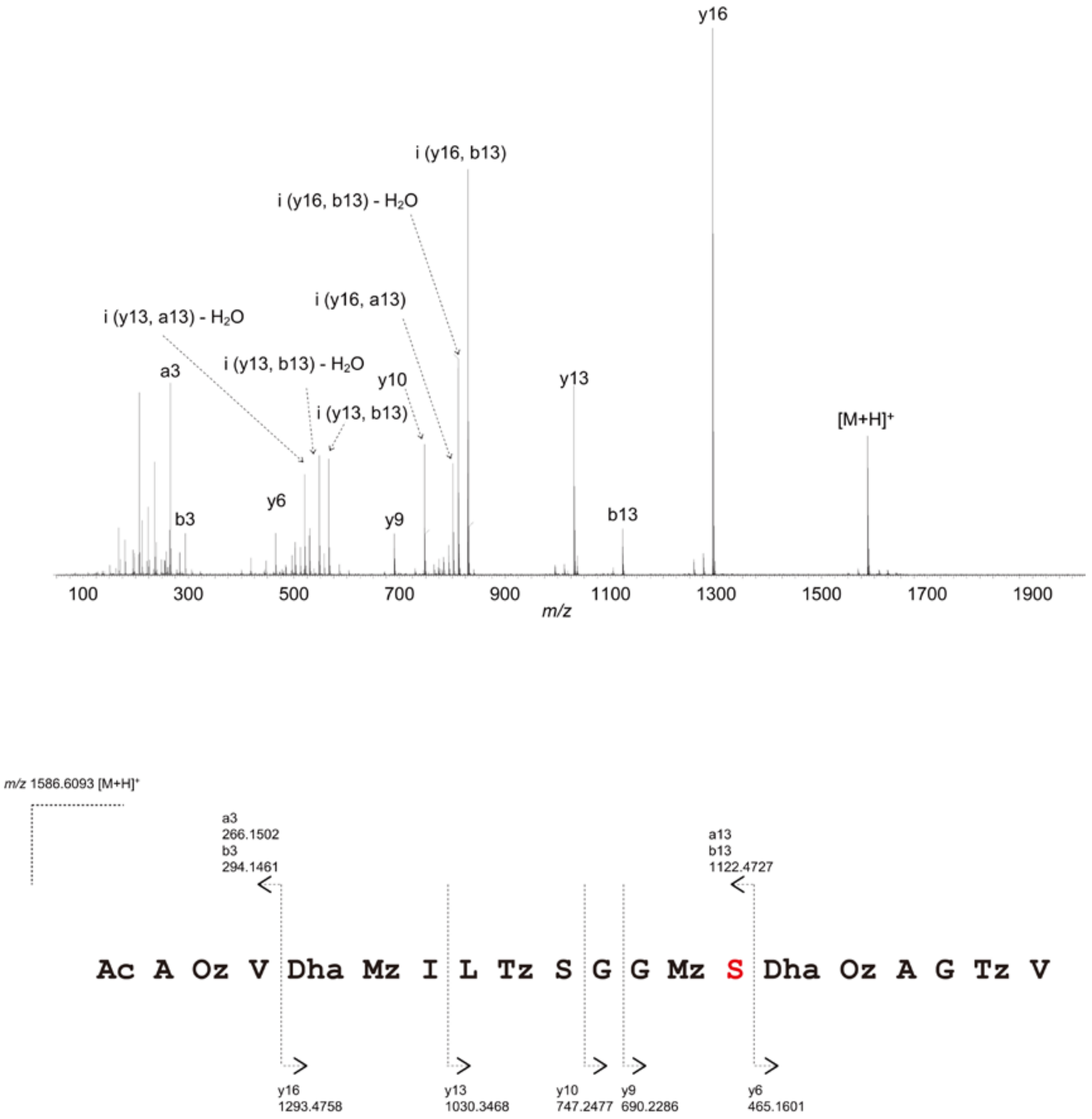

Supplementary Figure 25. MS fragmentation analysis of L13S. MS^E spectrum and assignment for fragmentation are shown. The labels, i (ym, a/bn) indicate the internal peptide fragment derived from fragments ym and a/bn.

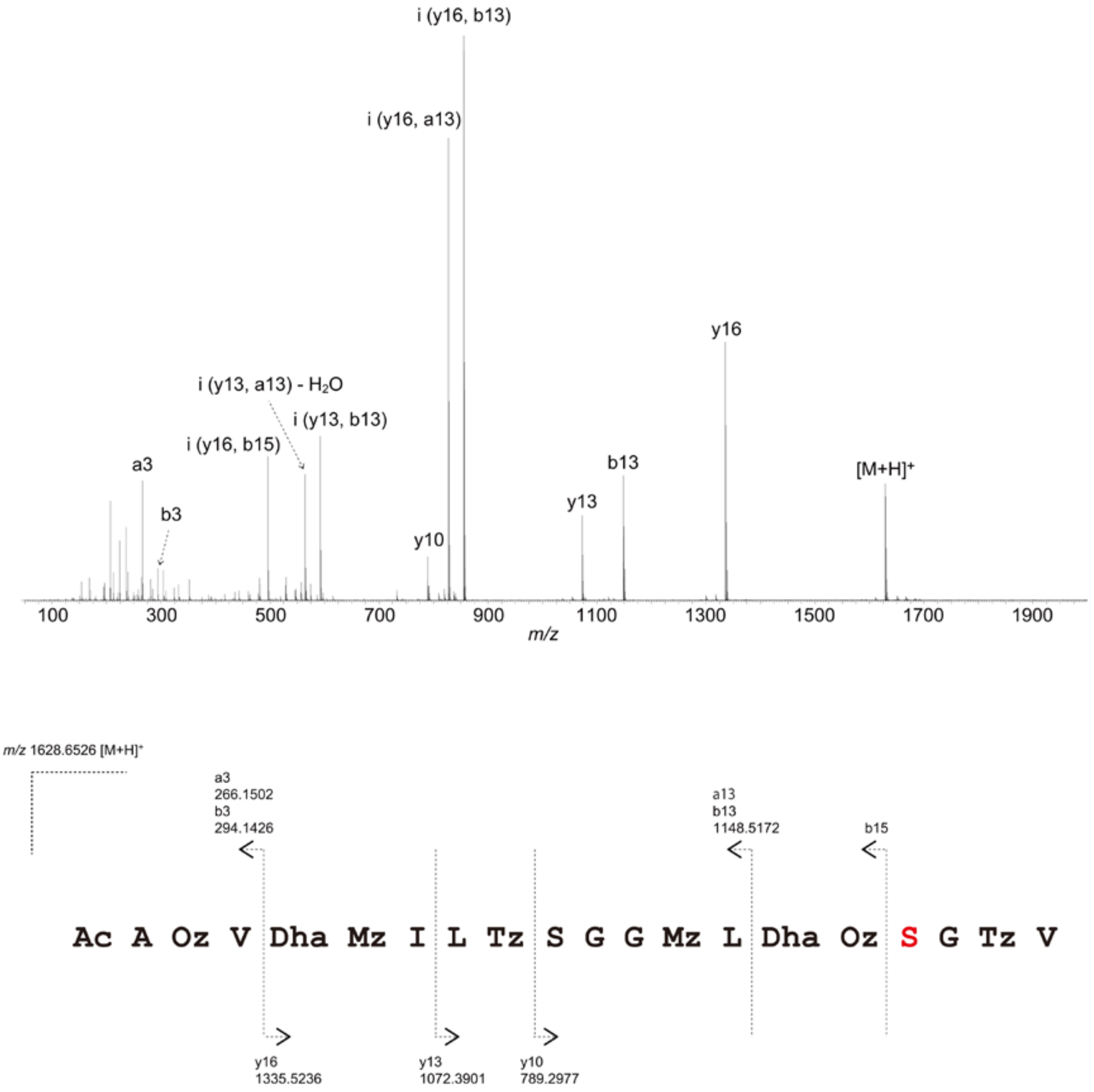

Supplementary Figure 26. MS fragmentation analysis of A16S. MS^E spectrum and assignment for fragmentation are shown. The labels, i (ym, a/bn) indicate the internal peptide fragment derived from fragments ym and a/bn. The fragment, i (y13, b15) was observed as a divalent ion.

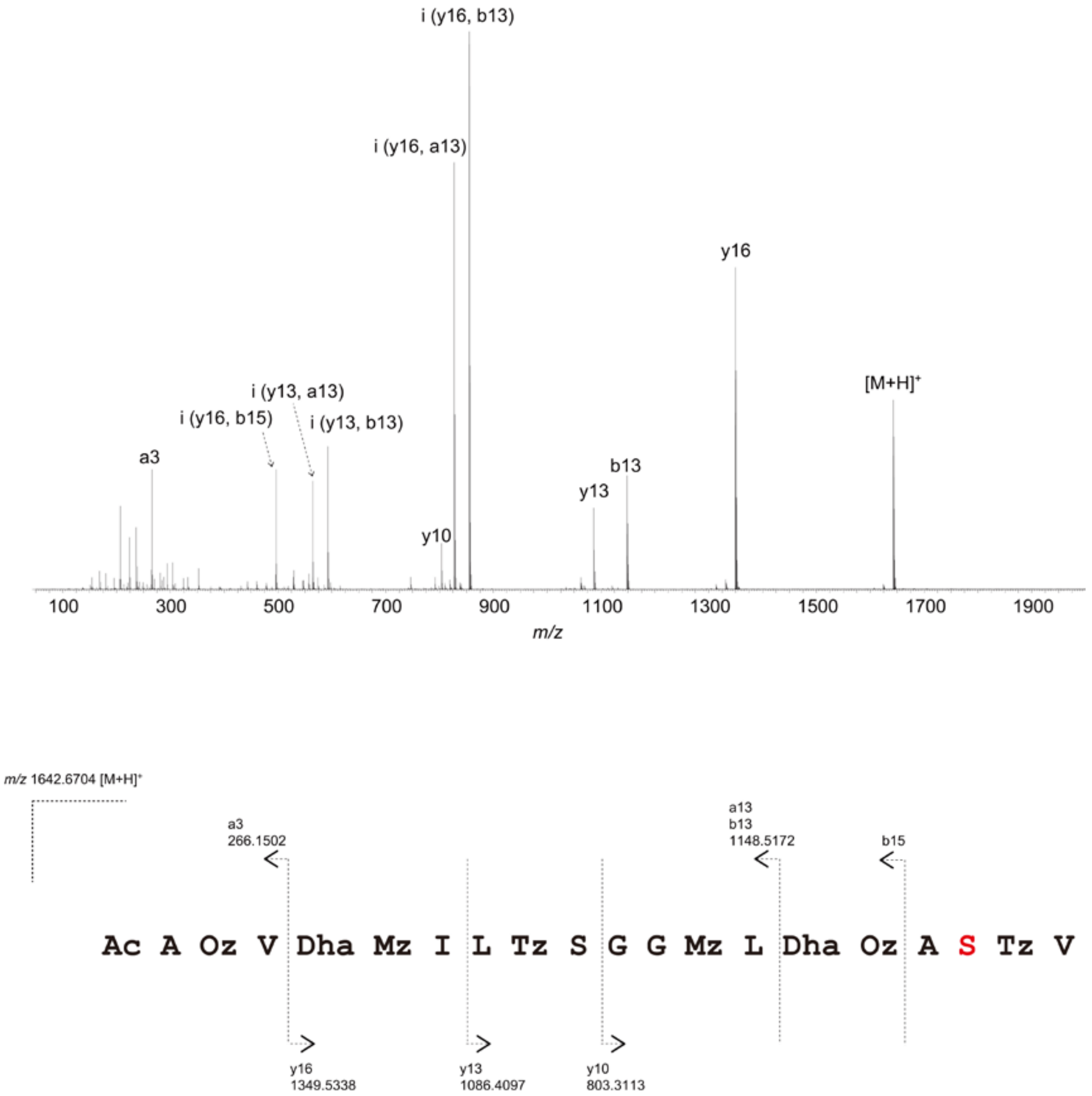

Supplementary Figure 27. MS fragmentation analysis of G17S. MS^{E} spectrum and assignment for fragmentation are shown. The labels, i (ym, a/bn) indicate the internal peptide fragment derived from fragments ym and a/bn. The fragment, i (y16, b15) was observed as a divalent ion.

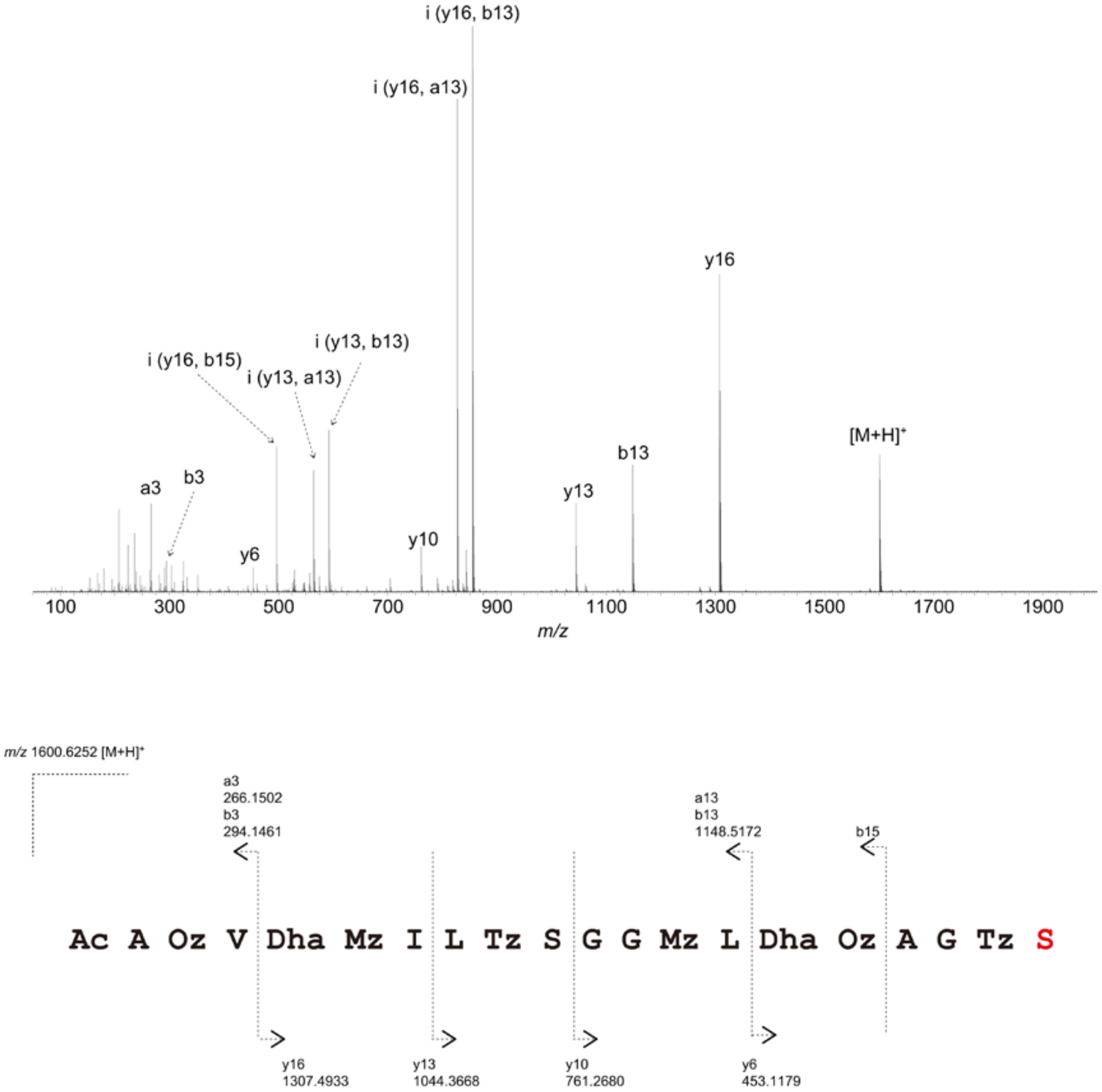

Supplementary Figure 28. MS fragmentation analysis of V19S. MS^{E} spectrum and assignment for fragmentation are shown. The labels, i (ym, a/bn) indicate the internal peptide fragment derived from fragments ym and a/bn. The fragment, i (y16, b15) was observed as a divalent ion.

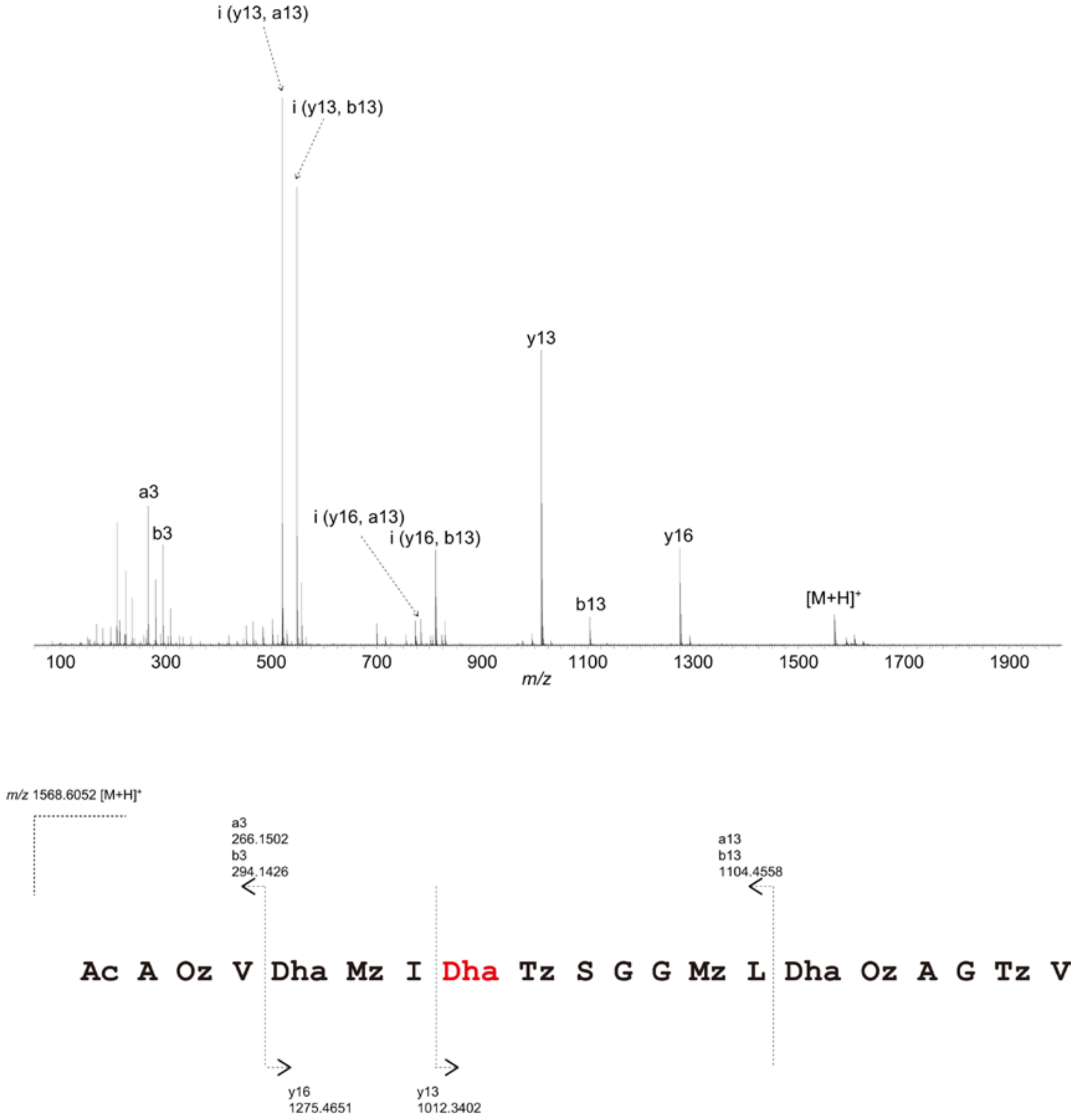

Supplementary Figure 29. MS fragmentation analysis of the minor product of L7S.

MS^E spectrum and assignment for fragmentation are shown. The labels, i (ym, a/bn) indicate the internal peptide fragment derived from fragments ym and a/bn.

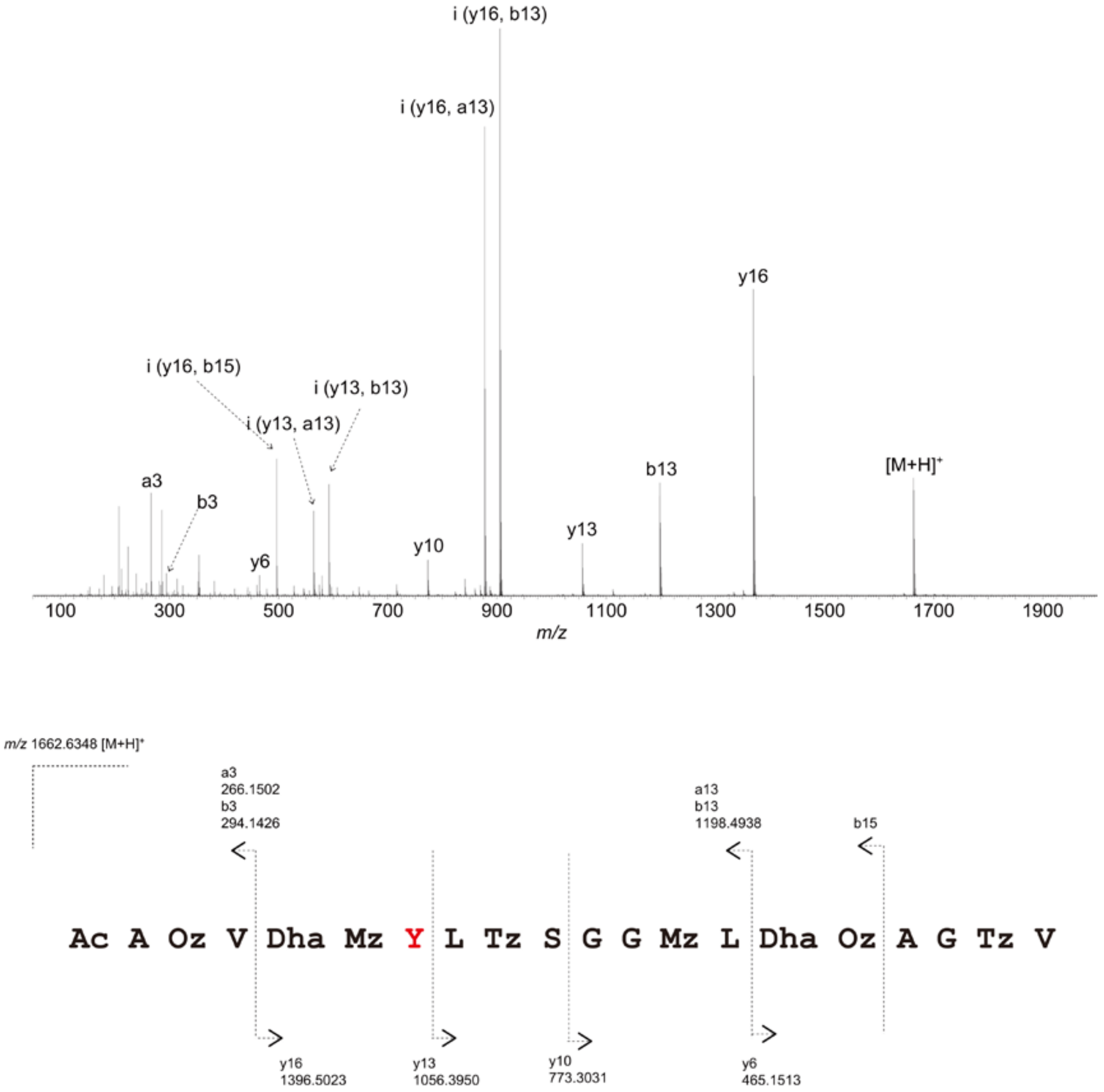

Supplementary Figure 30. MS fragmentation analysis of I6Y. MS^{E} spectrum and assignment for fragmentation are shown. The labels, i (ym, a/bn) indicate the internal peptide fragment derived from fragments ym and a/bn. The fragment, i (y16, b15) was observed as a divalent ion.

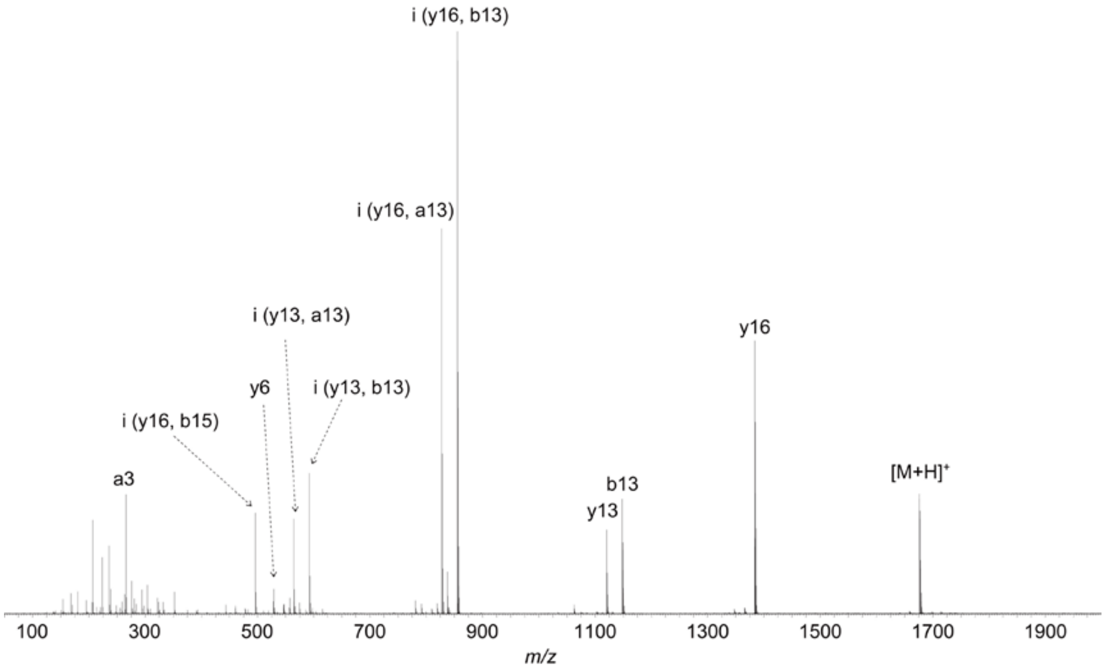

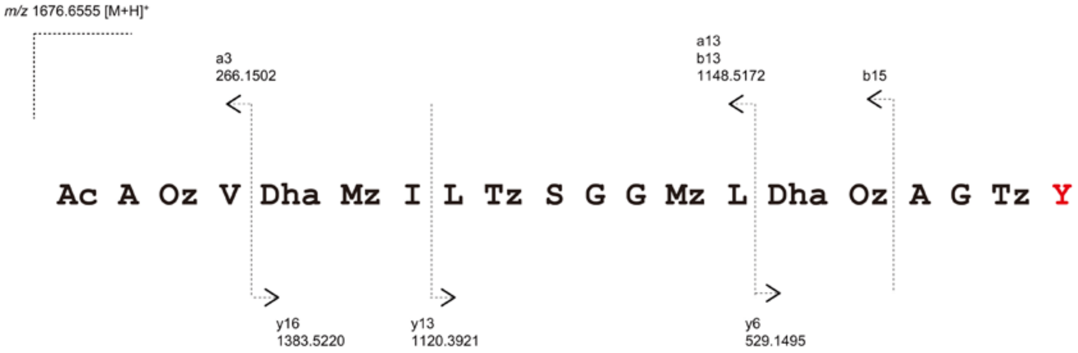

Supplementary Figure 31. MS fragmentation analysis of V19Y. MS^E spectrum and assignment for fragmentation are shown. The labels, i (ym, a/bn) indicate the internal peptide fragment derived from fragments ym and a/bn. The fragment, i (y16, b15) was observed as a divalent ion.

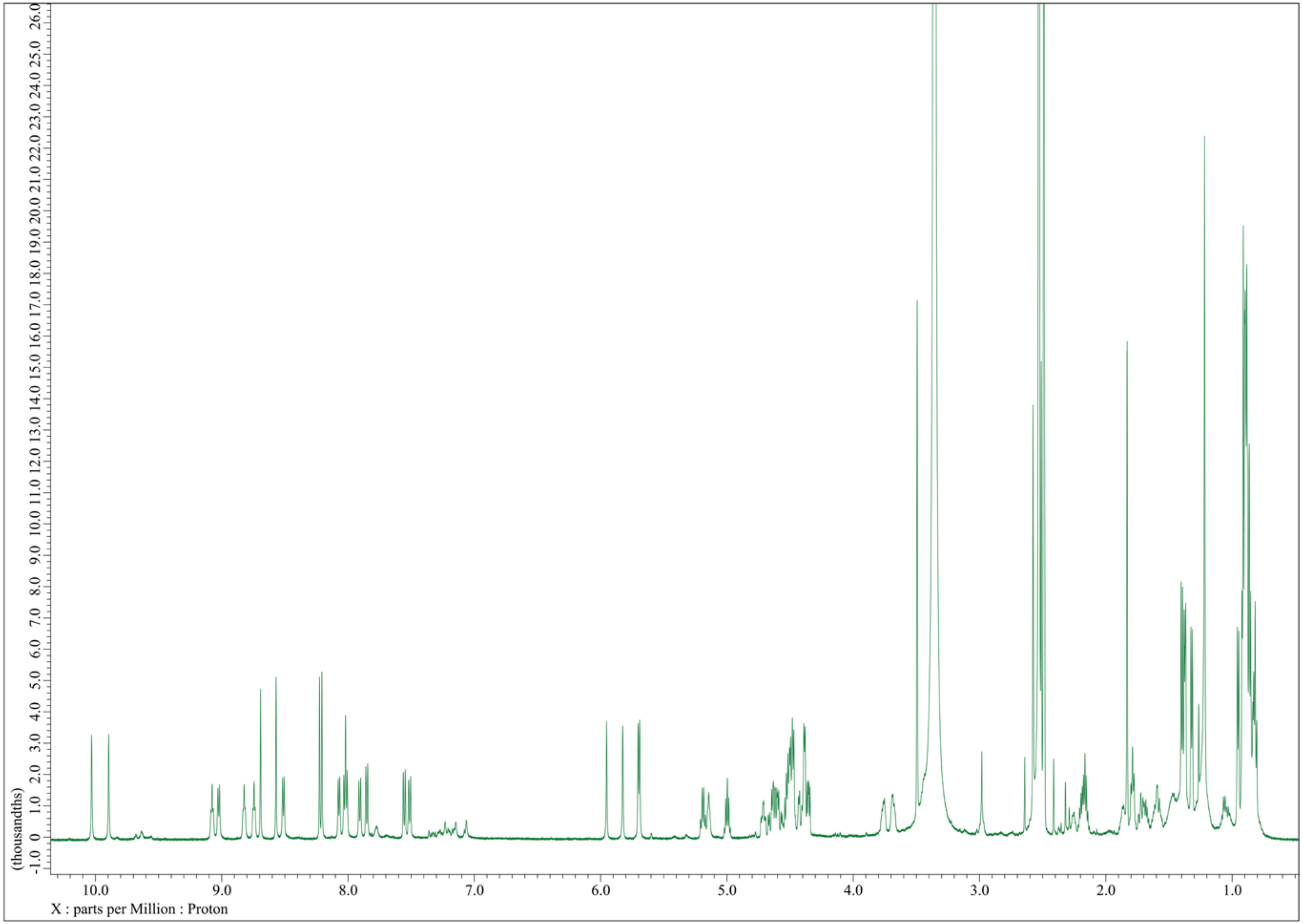

Supplementary Figure 32. ^{1}**H NMR spectrum of GS-10SA11 (DMSO-*d*$_6$, 600 MHz)**

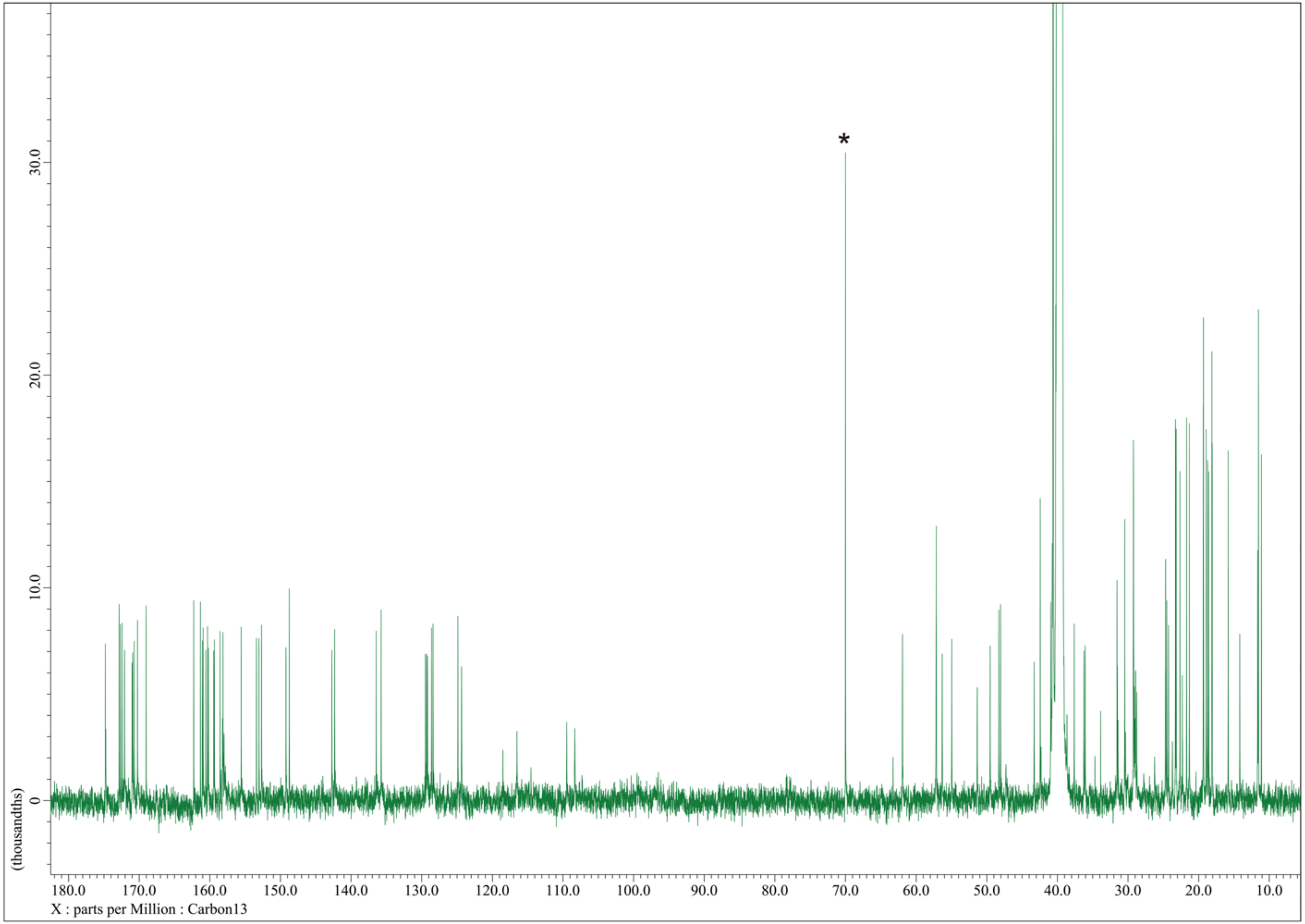

Supplementary Figure 33. ^{13}C NMR spectrum of GS-10SA11 (DMSO-d_6, 150 MHz). Asterisk indicates the signal derived from impurity.

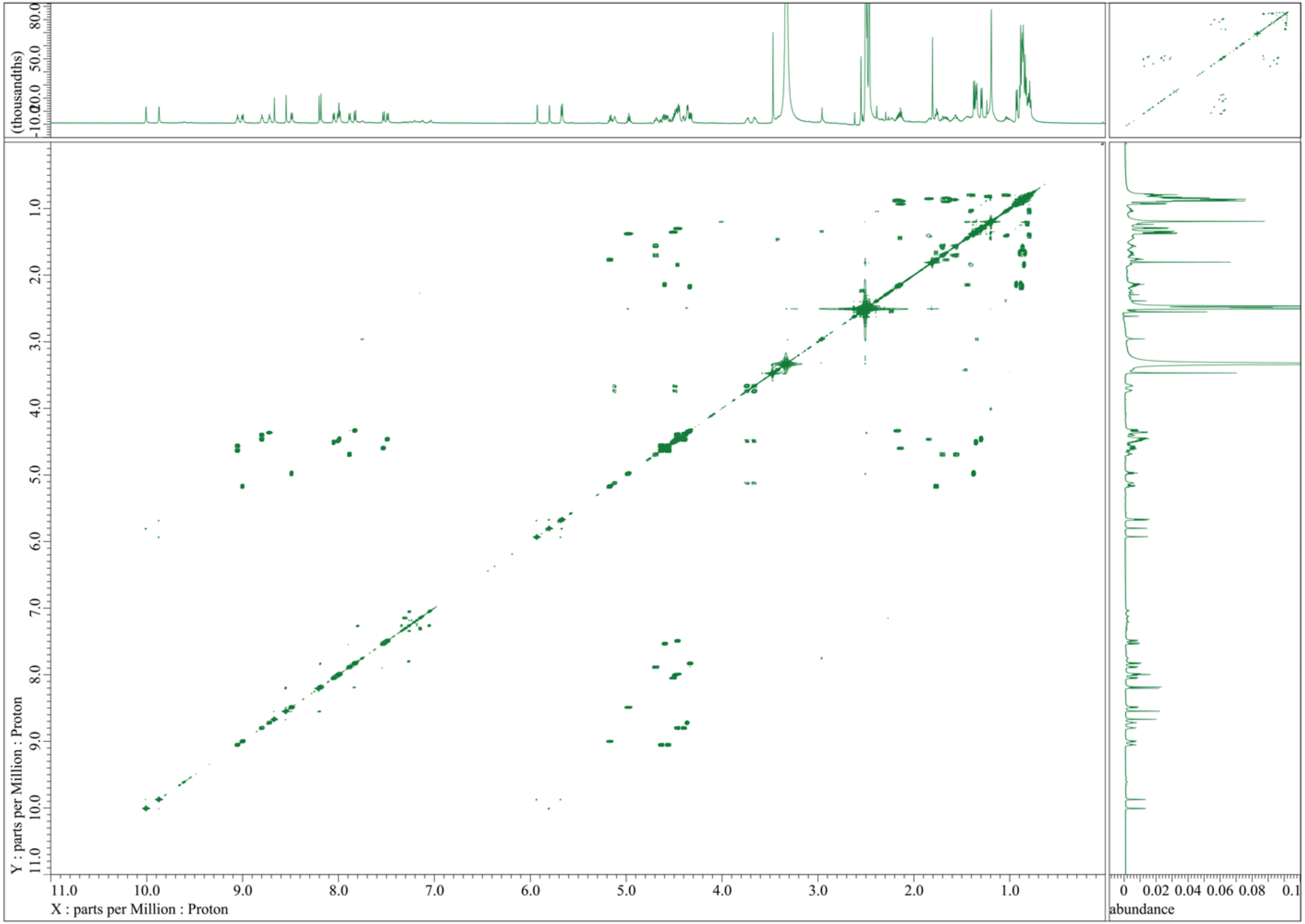

Supplementary Figure 34. COSY spectrum of GS-10SA11 (DMSO-*d_6*)

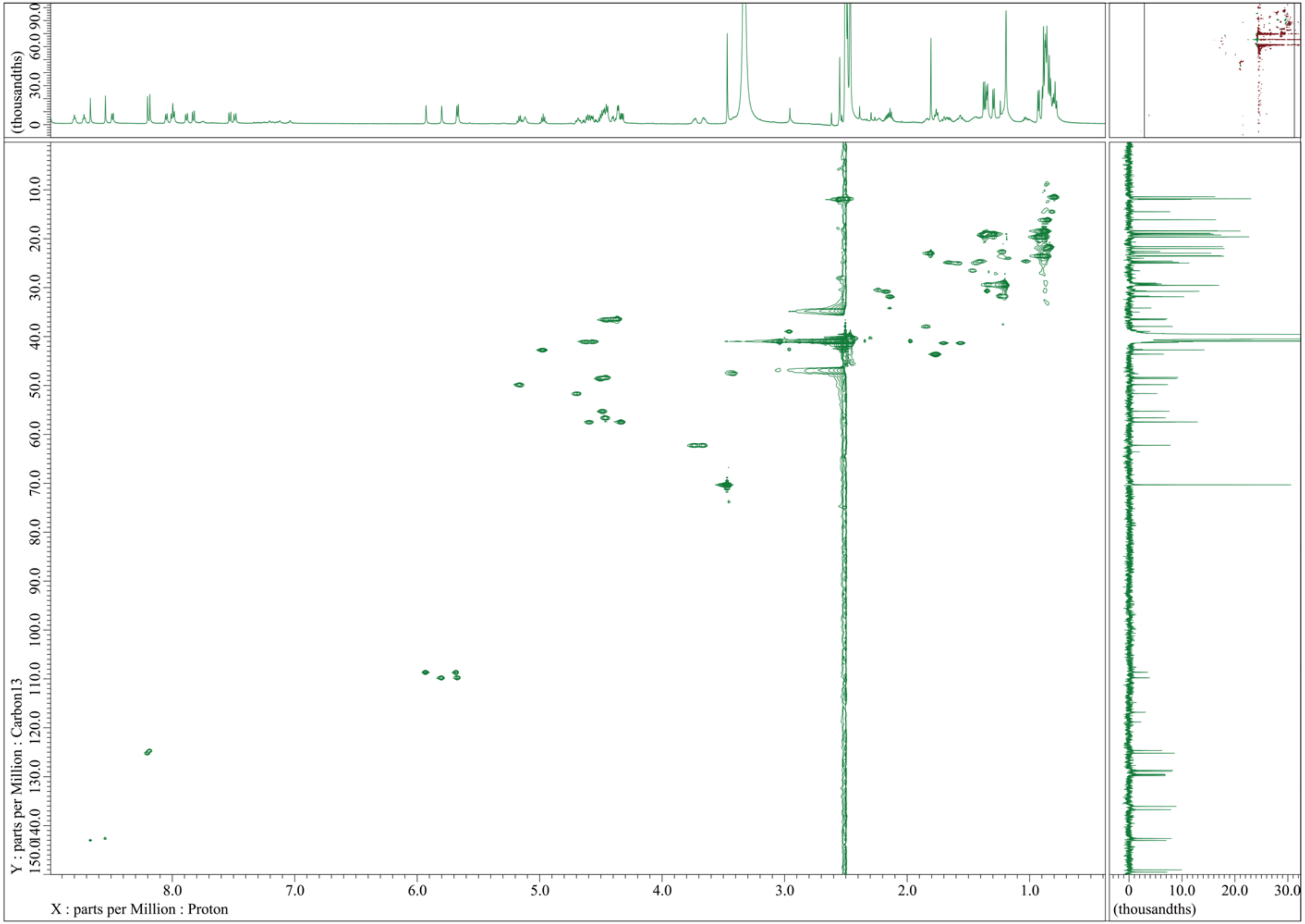

Supplementary Figure 35. HSQC spectrum of GS-10SA11 (DMSO-d_6)

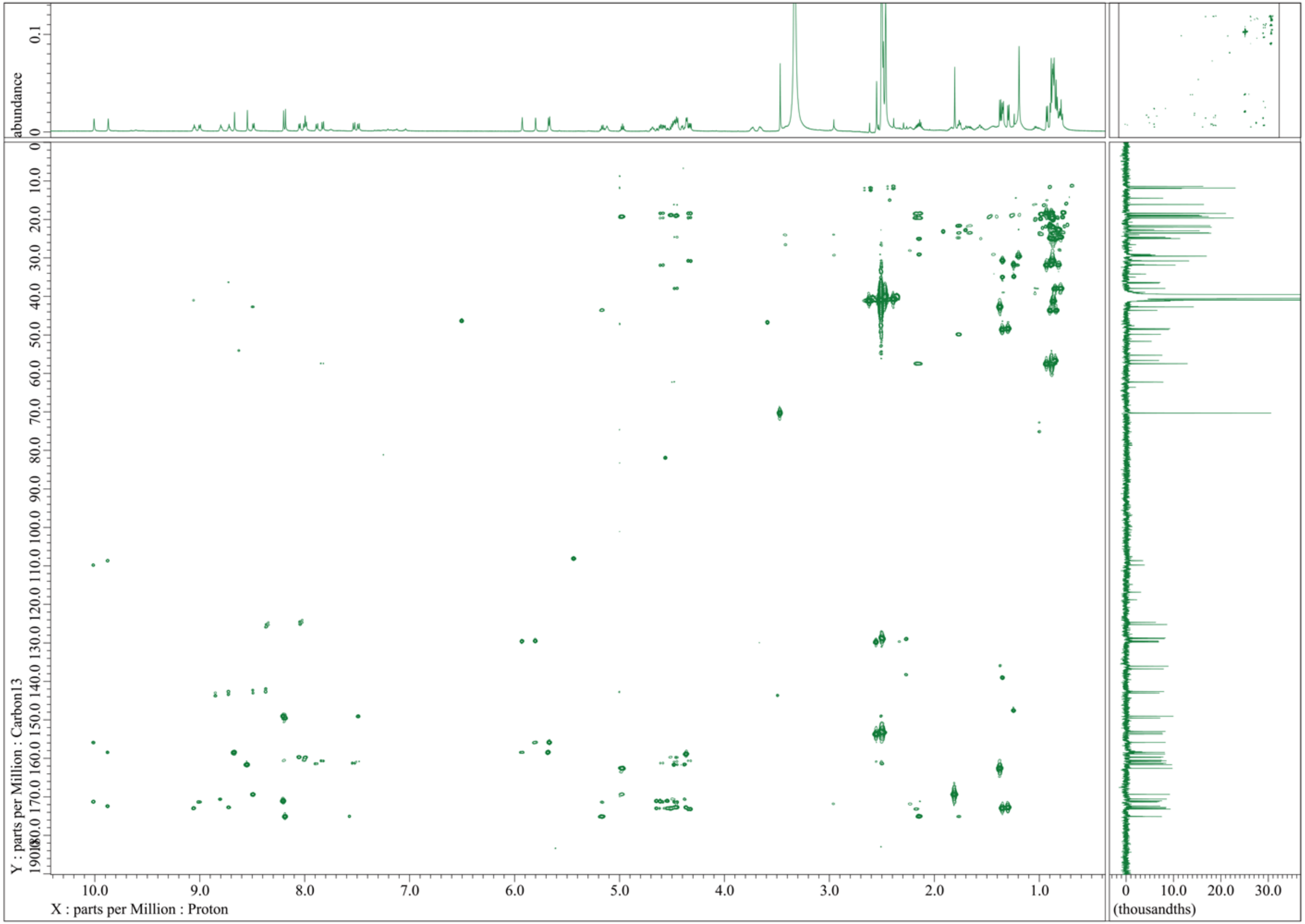

Supplementary Figure 36. CT-HMBC-1[1] spectrum of GS-10SA11 (DMSO-d_6)

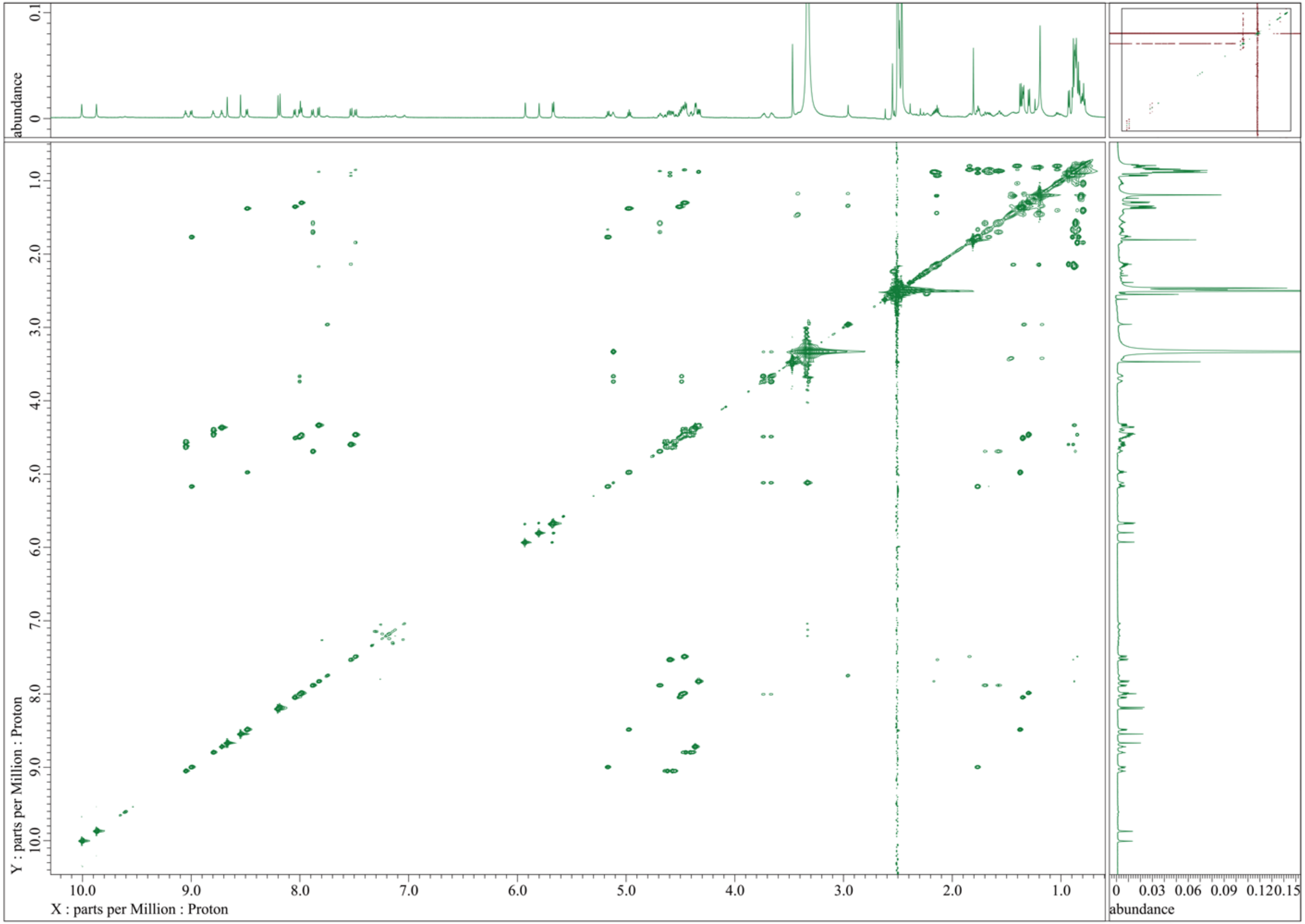

Supplementary Figure 37. TOCSY spectrum of GS-10SA11 (DMSO-d_6)

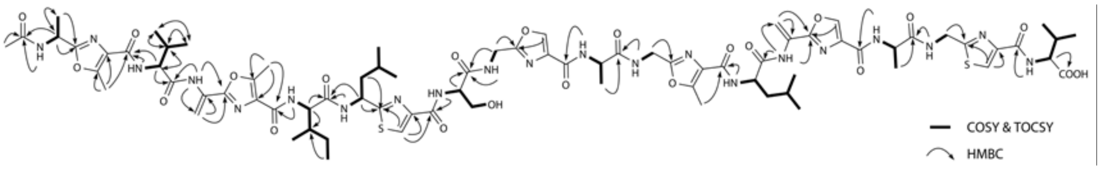

Supplementary Figure 38. Correlations observed in 2D NMR analyses of GS-10SA11. Bold lines indicate correlations in COSY and TOCSY analyses. Arrows indicate correlations in CT-HMBC-1.

Supplementary Table 1. Summary of NMR analysis of GS-10SA11 (DMSO-d6).

No.	residue		δ_C	δ_H (m, *J* in Hz)
1	AcAla	NH	-	8.51 (d,7.8)
		Ca	42.43	5.00 (quint, 7.8)
		Cb	18.94	1.40 (d, 7.2)
		Ac-CO	169.03	-
		Ac-Me	22.64	1.83 (s)
2	Mz	2	162.29	-
		4	128.39	-
		5	152.69	-
		5-Me	11.52	2.53 (s)**
		C=O	160.95	-
3	Val	NH	-	7.55 (d, 9.0)
		C=O	170.92	-
		Ca	57.18	4.62 (dd, 6.6, 9.0)
		Cb	31.56	2.16 (m)
		Cg	19.34	0.95 (d, 6.6)
		Cg	18.07	0.92 (m)
4	Dha	NH	-	10.03 (s)
		Ca	129.18	-
		Cb	109.47	5.83 (s)
				5.69 (s)
5	Mz	2	155.56	-
		4	129.48	-
		5	153.4	-
		5-Me	11.64	2.58 (s)
		C=O	160.56	-
6	Ile	NH	-	7.52 (d, 9.6)
		C=O	171.02	-
		Ca	56.31	4.5 (m)
		Cb	37.62	1.86 (m)
		Cg	24.27	1.42 (m)
				1.06 (m)
		Cg	15.81	0.88 (m)

		Cδ	11.11	0.82(t, 7.2)
7	Leu	NH	-	9.02 (d, 8.4)
		Ca	49.53	5.19 (q, 7.5)
		Cb	43.29	1.79 (t, 7.2)
		Cg	24.51	1.68 (m)
		Cd	23.18	0.92 (m)
		Cd	21.32	0.86 (d, 7.2)
8	Tz	2	174.8	-
		4	149.23	-
		5	124.37	8.21 (s)
		C=O	160.2	
9	Ser	NH	-	8.03 (d, 7.8)
		C=O	170.25	-
		Ca	54.96	4.5 (m)
		Cb	61.94	3.76 (m)
				3.68 (m)
		-OH	-	5.14 (brt. 4.8)
10	Gly	NH	-	8.82 (t, 6.0)
		Ca	36.25	4.42 (dd, 5.4, 16.8)
				4.5 (m)
11	Oz	2	161.34	
		4	136.44*	
		5	142.33	8.57 (s)
		C=O	159.45	
12	Ala	NH	-	8.01 (d, 8.4)
		C=O	172.41	-
		Ca	48.05	4.5 (m)
		Cβ	18.73	1.32 (d, 7.2)
13	Gly	NH	-	8.75 (t, 5.4)
		Ca	36.07	4.39 (d, 6.0)
14	Mz	2	158.57	-
		4	128.6	-
		5	153.04	-
		5-Me	11.52	2.51 (s)
		C=O	161.06	-

15	Leu	NH	-	7.91 (d, 9.0)
		C=O	172.08	-
		Ca	51.36	4.71 (td, 4.2, 9.0)
		Cβ	40.93	1.72 (m)
				1.59 (m)
		Cg	24.67	1.6 (m)
		Cd	23.28	0.89 (m)
		Cd	21.71	0.89 (m)
16	Dha	NH	-	9.90 (s)
		Ca	129.3	-
		Cβ	108.34	5.95 (s)
				5.70 (s)
17	Oz	2	158.14	-
		4	135.75*	-
		5	142.72	8.69 (s)
		C=O	159.35	-
18	Ala	NH	-	8.07 (d, 7.2)
		C=O	172.65	-
		Ca	48.29	4.5 (m)
		Cβ	18.57	1.37 (d, 7.2)
19	Gly	NH	-	9.08 (t, 6.0)
		Ca	40.73	4.65 (dd, 6.0, 16.8)
				4.58 (dd, 7.2, 16.2)
20	Tz	2	170.71	-
		4	148.75	-
		5	124.89	8.23 (s)
		C=O	160.33	-
21	Val	NH	-	7.85 (d, 9.0)
		C=O	172.84	-
		Ca	57.15	4.35 (dd, 5.4, 9.0)
		Cβ	30.46	2.19 (m)
		Cg	18.12	0.89 (m)
		Cg	19.31	0.91 (m)

*: These signals are exchangeable. Assignments were based on chemical shifts in the

literature because no correlations were observed in 2D NMR spectra.

**: This signal was overlapped by the signal derived from residual DMSO.

Supplementary Table 2. Observed and calculated *m/z* of designer GS analogs produced *in vivo*, and estimated chemical formula of monoisotopic ions.

Name	Observed	Calculated	Chemical formula
GS-10SA11	1750.7136 $[M+H]^+$	1750.7101 $[M+H]^+$	C78 H104 N21 O22 S2
GS-tandem	1582.6585 $[M+2H]^{2+}$	1582.6566 $[M+2H]^{2+}$	C142 H192 N38 O38 S4

Supplementary Table 3. Sequences of *godD* and *godA-tandem*.

gene	Sequence (shown from 5’ to 3’)
godD	ATGGCACCGGTGTTACACTTTCGCCGCAGCTTTAAGGTGGACCTG<br>CTGCAGGGTGACGGTGTTTACCTGACCAGTGATCGTGGTGAGAGC<br>ACAGTGCTGCGTGGTCAGCTGGTTGAGAGCTTAAGTCCGCTGCTG<br>ATGGCAGGCCGCACAGAAGATGATCTGGTTACCGCCCTGGCAGGT<br>ATGTTCCCTGCACAGCGCATCTTAAGCGCACTGCGCCAACTGGAA<br>AAAGCAGGCTACGTTCGCCGTGCCGAGGCAGCAACCGGTGCAGA<br>GACCTTTGCCGCAGGTTTCTGGGAGAGCAGTGGTGTGGACGGCA<br>GTACCGCACTGGCCCGTCTGGCAAGCCGTACCGTTCGTATTACAG<br>GTGTTGGCCATGCCGGCAGCGAAGGTGATGTGGTTCGTCGTGTGC<br>GCAAAGCAGGTAGCAGTCTGGGCCTGCATCTGACCGACGGCAGC<br>GCAGACCTGACAGTGGTTGTGACCGTGGACTATCTGGAGCCGGAA<br>CTGGCCCGCATCAACGCAGAGGCCTTAGCCGATGGCCGCCCGTG<br>GATGCTGGCAAAACCTGGCGGTAGCGTTGCCTGGTTTGGTCCGTT<br>CTTCCGCCCGGGCGAAAGCGCATGCTGGGCCTGTCTGGCCCATC<br>GTCTGAGCGGCAACCGCATGATCGAAACCTATGTGGCCAAAGCAC<br>CGACACGCAGCGCAAGCTATAGCCCGACCGTGAACCTGCCGGCC<br>ACACAAACCGCAGCAGAGGAGTTAACCGCCCTGCACGCAGCCAAA<br>TGGTTAGCAGGCGTTTATGCCGCCCAGGCCAGTGAACACAGTGCA<br>CGCCCGCAGGGTCTGAATCCGGATCTGCTGCACACATTCGATGCA<br>GTTACCTTAGCCGCACAGGAGCACCTGGTGTTTCGTCGTCCGCAG<br>TGCCCTGAGTGTGGTGATGGCGAGTTAATGGCCCGCCAGCATCAG<br>AGTCCGGTTCGTCTGCGCAGCATGCCGAAGGTTACACTGGTGGAC<br>GGTGGTCATCGTAGCAAAGACCCGCAGCGTATGCTGGACCTGCAT<br>GGCCACCTGATTAGCAGCGCCCTGGGTCCGGTTACCGGCTTACAG<br>AAGGTTCCGAGCGTTTGGCCTGGCTTCCATGCCTATACCGCCGGT<br>CAAAATTTCGCCATCCCGATGAGCCGTCCTGGTGATTTACGTGTGG<br>GCCTGCGTAGTCAGAGCTGCGGCAAAGGTATGAGCGATTTACAGG<br>CCCGCGCAAGTGCACTGGGTGAAGCCCTGGAGCGTTACAGTGGT<br>GTTTATCAGGGCGACGAAGCACGCATTACCGCAAGTTACGACGATC<br>TGGGCGATCGCGCAATCGCCCCGAACGATTTAGCCCTGTATAGTGC<br>CCGCCAGTTCGACGAACGCGAAGAGTGGAACAACCGCGACGTTC<br>ATTTCCACCGTGTGCTGGCCCCTTTCGACACCGCAGCACCGATTG<br>ACTGGACCCCGGTGTGGAGTCTGACCATGCAGCGCCATCGCTATG<br>TTCCTACCGCCAGCCTGTTCTACGGTTACCCTCTGGATCGCGACCA

	TCAGTATGCAGCCGCCGATAGCAATGGCAGCGCAGCCGGTACCAG<br>CATCGAGGACGCAGTGCTGCAAGGTTTTATGGAGCTGGTTGAGCG<br>TGACAGTGTGGCCCTGTGGTGGTATAACCGTGTGCAACGTCCTGA<br>AGTGGATCTGCAGAGTTTCGGCGAGCCGTATTTTCTGGAGTGGCT<br>GGCCCAATATCGCAGTCTGAATCGTGAGGCATGGGTGCTGGATCT<br>GACCAGCGATTTCGGTATCCCGGTGATGGCAGCCATTAGCCGTCG<br>TATCGACAAGCCGGCCGAAGACATCCTGATCGCATTCGGTGCACA<br>CTTTGACGCCCGTATTGCCGTTGGTCGTGCCCTGACAGAGATGAA<br>CCAATTCCTGCCGGCCGTTGTTCACGCCAAACCGGAAGGTGGTGG<br>TTATACATACCCGGATCCGGCCCAGCAGCATTGGTGGCAAACCGCC<br>ACACTGGCCAACCAACCTTATCTGCGCCCGCTGAGTGCACCGCGT<br>CGTACCGCAGGTGACTTCCCTGTGCACGAAAGTCTGGATCTGCTG<br>GATGATCTGCATCGCGCACAAGCAACCGTGGAAGAGCACGGCATG<br>GAGCTGCTGGTGATCAATCAGACCCGCCCGGACGTGGGTCTGCC<br>GGTTGTTAAGGTGATTGTGCCTGGCATGCGTCACTTCTGGCCGCG<br>TTTCGCCCCGGGTCGCTTATACGATGTGCCGGTGAAACTGGGCTG<br>GGTTAGCCAGCAGACCCGCGAAGAGGACCTGAATCCGATTGGCGT<br>GTTTATTTGA
godA-tandem	ATGGAGAACGTCCAGACCCTGGCGATCGACGACATCGAGAACATC<br>GACGCTGAGGTGACCATCGAGGAGCTTTCCTCGACCAACGGCGC<br>CGCCACCGTCAGCACCATCCTGTGCAGCGGCGGCACCCTCAGCT<br>CGGCCGGCTGCGTCGCCACCGTCAGCACCATCCTGTGCAGCGGC<br>GGCACCCTCAGCTCGGCCGGCTGCGTCTGA

Supplementary Table 4. Primers used for gene cloning. Restriction sites are underlined and corresponding restriction enzymes are shown in parentheses.

Name	Sequence (restriction sites were underlined)
pgodDopt-F-NdeI	GGGGGGCATATGGCACCGGTGTTAC (*Nde*I)
pgodDopt-R-XhoI	GGGCTCGAGTTAAATAAACACGCCAATCGG (*Xho*I)
godE-N-Nde	GGTGTTCATCATATGATTGTCTCCGACACG (*Nde*I)
godE-Cter-Hind	CGGGCGAAGCTTTTGGGACTCGTCCTCCAG (*Hin*dIII)
godF-N-Nde	GGACGAGTCCATATGACCTCGCCCGCCGCA (*Nde*I)
godF-C-Xho	GATTCGCTCGAGTCAGACAAATGCCACCAG (*Xho*I)
lazF-NdeI	GGGGGGCATATGACCACCCACGCG (*Nde*I)
lazF-R-XhoI	GGGCTCGAGCTCCATGATCACCG (*Xho*I)
godH-N-Nde	GAAGGATTCCATATGAATGAGATCACCTGG (*Nde*I)
godH-C-Xho	TGCGTTCTCGAGTTATGACTGAAGTGAAAC (*Xho*I)

Supplementary Table 5. Primers used for the syntheses of DNA templates for *in vitro* translation.

No.	Name	Sequence (restriction site)
1	godA*-F1	GGCGTAATACGACTCACTATAGGGTTAACTTTAACAAGGAGAAAAACAT
2	godA*-F2	GGCGTAATACGACTCACTATAG
3	godA*-R1	GATATTCTCAATATCGTCAATTGCCAGAGTTTGCACGTTTTCCATGTTTTTCTCCTTGTT
4	godA*-R2	ACCATTAGTAGAGCTCAGTTCCTCAATCGTAACCTCAGCGTCGATATTCTCAATATCGTC
5	godA*-R3	CCCCCGCTACACAAGATCGTGCTCACGGTAGCTGCACCATTAGTAGAGCTCAG
6	godA*-R4	CGAAGCTTAAACGCAACCTGCAGAACTAAGAGTACCCCCGCTACACAAG
7	godA*truncate_R2	CACCATTGGTAGAGCTCAGTTCCTCAATGGTAACCTCAGCGTCGATATTCTCAATATCGTC
8	godA*1–9_R3	CGAAGCTTATGAACATAAGATGGTAGACACGGTAGCTTCTGCACCATTAGTAGAGCTCAG
9	godA*1–6-R3	CGAAGCTTAGATGGTAGACACGGTAGCTTCTGCACCATTAGTAGAGCTCAG
10	godA*1–3-R3	CGAAGCTTACACGGTAGCTTCTGCACCATTAGTAGAGCTCAG
11	godA*1–2-R3	CGAAGCTTAGGTAGCTTCTGCACCATTAGTAGAGCTCAG
12	godA*2–3-R3	CGAAGCTTACACGGTTTCTGCACCATTAGTAGAGCTCAG
13	godA*1–9T2A/T5A/C8A-R3	CGAAGCTTATGAAGCTAAGATAGCAGACACTGCAGCTTCTGCACCATTAGTAGAGCTCAG
14	godA*1–6-T2A/T5A-R3	CGAAGCTTAGATAGCAGACACTGCAGCTTCTGCACCATTAGTAGAGCTCAG
15	godA*1–3-T2A -R3	CGAAGCTTACACTGCAGCTTCTGCACCATTAGTAGAGCTCAG
16	godA*1–8-R3	CGAAGCTTAACATAAGATGGTAGACACGGTAGCTTCTGCACCATTAGTAGAGCTCAG
17	godA*2–9-R3	CGAAGCTTATGAACATAAGATGGTAGACACGGTTTCTGCACCATTAGTAGAGCTCAG
18	godA*1–5-R3	CGAAGCTTAGGTAGACACGGTAGCTTCTGCACCATTAGTAGAGCTCAG
19	godA*2–6-R3	CGAAGCTTAGATGGTAGACACGGTTTCTGCACCATTAGTAGAGCTCAG
20	godA*1–3-A1C/T2A-R3	CGAAGCTTACACAGCACATTCTGCACCATTAGTAGAGCTCAG

21	godA*1–3-A1T/T2A-R3	CGAAGCTTACACAGCGGTTTCTGCACCATTAGTAGAGCTCAG
22	godA*1–3-A1S/T2A-R3	CGAAGCTTACACAGCAGATTCTGCACCATTAGTAGAGCTCAG
23	godA*1–3-T2A/V3C-R3	CGAAGCTTAACATGCAGCTTCTGCACCATTAGTAGAGCTCAG
24	godA*1–3-T2A/V3T-R3	CGAAGCTTAGGTTGCAGCTTCTGCACCATTAGTAGAGCTCAG
25	godA*1–3-T2A/V3S-R3	CGAAGCTTAAGATGCAGCTTCTGCACCATTAGTAGAGCTCAG
26	godA*1–9-A1T/T2A-R3	CGAAGCTTATGAACATAAGATGGTAGACACAGCGGTTTCTGCACCATTAGTAGAGCTCAG
27	godA*1–6-A1T/T2A -R3	CGAAGCTTAGATGGTAGACACAGCGGTTTCTGCACCATTAGTAGAGCTCAG
28	godA*1–6-T5I/I6T-R3	CGAAGCTTAGGTGATAGACACGGTAGCTTCTGCACCATTAGTAGAGCTCAG
29	godA*1–9-T2V/V3T-R3	CGAAGCTTATGAACATAAGATGGTAGAGGTCACAGCTTCTGCACCATTAGTAGAGCTCAG
30	godA*1–6-T2V/V3T -R3	CGAAGCTTAGATGGTAGAGGTCACAGCTTCTGCACCATTAGTAGAGCTCAG
31	godA*1–9-L7C/C8L-R3	CGAAGCTTATGATAAACAGATGGTAGACACGGTAGCTTCTGCACCATTAGTAGAGCTCAG
32	godA*1–9-$_{-1}A_{1}$-R3	CGAAGCTTATGAACATAAGATGGTAGACACGGTAGCTGCTTCTGCACCATTAGTAGAGCTCAG
33	godA*1–9-$_{3}A_{4}$-R3	CGAAGCTTATGAACATAAGATGGTAGATGCCACGGTAGCTTCTGCACCATTAGTAGAGCTCAG
34	godA*1–9-$_{6}$A7$_{-}$R3	CGAAGCTTATGAACATAATGCGATGGTAGACACGGTAGCTTCTGCACCATTAGTAGAGCTCAG
35	godA*1–3-T2C-R3	CGAAGCTTACACACAAGCTTCTGCACCATTAGTAGAGCTCAG
36	godA*1–3-T2S-R3	CGAAGCTTACACTGAAGCTTCTGCACCATTAGTAGAGCTCAG
37	godA*1–3-A1E-R3	CGAAGCTTACACGGTTTCTTCTGCACCATTAGTAGAGCTCAG
38	godA*1–3-A1R-R3	CGAAGCTTACACGGTACGTTCTGCACCATTAGTAGAGCTCAG
39	godA*1–3-A1Y-R3	CGAAGCTTACACGGTATATTCTGCACCATTAGTAGAGCTCAG
40	godA*1–3-A1W-R3	CGAAGCTTACACGGTCCATTCTGCACCATTAGTAGAGCTCAG
41	godA*1-3-V3R-R3	CGAAGCTTAACGGGTAGCTTCTGCACCATTAGTAGAGCTCAG
42	godA*1–3-V3Y-R3	CGAAGCTTAATAGGTAGCTTCTGCACCATTAGTAGAGCTCAG

43	godA*1–3-V3W-R3	CGAAGCTTACCAGGTAGCTTCTGCACCATTAGTAGAGCTCAG
44	godA*1–3-V3E-R3	CGAAGCTTATTCGGTAGCTTCTGCACCATTAGTAGAGCTCAG
45	godA*1–16-R3	CGAAGCTTATGCAGAACTAAGAGTACCCCCGCTACACAAG
46	godA*1–13-R3	CGAAGCTTAAAGAGTACCCCCGCTACACAAG
47	godA*T5A-R3	CCCCCGCTACACAAGATTGCGCTCACGGTAGCTTCTGCACCATTAGTAGAGCTCAG
48	godA*T15A-R4	CGAAGCTTAAACGCAACCTGCTGCACTAAGAGTACCCCCGCTACACAAG
49	godA*S4A-R3	CCCCCGCTACACAAGATCGTAGCCACGGTAGCTTCTGCACCATTAGTAGAGCTCAG
50	godA*S14A-R4	CGAAGCTTAAACGCAACCTGCAGATGCAAGAGTACCCCCGCTACACAAG
51	godA* LP(-25)–(-1)-R1	GATATTCTCAATATCGTCAATTGCCAGCTTTTTCTTCATGTTTTTCTCCTTGTT
52	godA* LP(-20)–(-1)-R1	GCGTCGATATTCTCAATCTTTTTCTTCATGTTTTTCTCCTTGTT
53	godA* LP(-20)–(-1)-R2	CACCATTAGTAGAGCTCAGTTCCTCAATCGTAACCTCAGCGTCGATATTCTCAAT
54	godA* LP(-15)–(-1)-R1	GTAGAGCTCAGTTCCTCAATCGTAACCTCAGCCTTTTTCTTCATGTTTTTCTCCTTGTT
55	godA* LP(-15)–(-1)-R2	CCCCCGCTACACAAGATCGTGCTCACGGTAGCTTCTGCACCATTAGTAGAGCTCAGTTCCTCAAT
56	godA* LP(-10)–(-1)-R1	CCATTAGTAGAGCTCAGTTCCTCCTTTTTCTTCATGTTTTTCTCCTTGTT
57	godA* LP(-10)–(-1)-R2	CCCCCGCTACACAAGATCGTGCTCACGGTAGCTTCTGCACCATTAGTAGAGCTCAG
58	godA* LP(-6)–(-1)-R1	GCTTCTGCACCATTAGTAGACTTTTTCTTCATGTTTTTCTCCTTGTT
59	godA* LP(-6)–(-1)-R2	GAGTACCCCCGCTACACAAGATCGTGCTCACGGTAGCTTCTGCACCATTAGT
60	godA* LP(-6)–(-1)-R3	CGAAGCTTAAACGCAACCTGCAGAACTAAGAGTACCCCCGCTACA
61	godA*-10SA11-R3	GACCCAGAACACAAGATCGTTGACACGGTAGCTTCTGCACCATTAGTAGAGCTCAG
62	godA*-10SA11-R4	CGAAGCTTAAACGCAACCTGCAGATGAAAGAGTACCAGCTGACCCAGAACACAAG
63	godA*-tandem-R3	GTACCCCCGCTACATAAGATCGTGCTCACGGTAGCTTCTGCACCATTAGTAGAGCTCAG
64	godA*-tandem-R4	CAGAATGGTAGAAACAGTTGCAACGCAACCTGCAGAACTAAGGGTACCCCCGCTACAT

Supplementary Table 6. List of primers used for the synthesis of DNA templated for GodA derivatives. Primers are indicated by the numbers in Supplementary Table 5.

	extension		1st PCR		2nd PCR		3rd PCR		4th PCR	
Name	Forward primer	Reverse primer	Forward primer	Reverse primer	Forward primer	Reverse primer	Forward primer	Reverse primer	Forward primer	Reverse primer
GodA*	1	3	2	4	2	5	2	6	N/A	
GodA*1–9	1	3	2	7	2	8	N/A		N/A	
GodA*1–6	1	3	2	7	2	9	N/A		N/A	
GodA*1–3	1	3	2	7	2	10	N/A		N/A	
GodA*1–2	1	3	2	7	2	11	N/A		N/A	
GodA*2–3	1	3	2	7	2	12	N/A		N/A	
GodA*1–9/T2A/T5A/C8A	1	3	2	7	2	13	N/A		N/A	
GodA*1–6-T2A/T5A	1	3	2	7	2	14	N/A		N/A	
GodA*1–3-T2A	1	3	2	7	2	15	N/A		N/A	
GodA*1–8	1	3	2	7	2	16	N/A		N/A	
GodA*2–9	1	3	2	7	2	17	N/A		N/A	
GodA*1–5	1	3	2	7	2	18	N/A		N/A	
GodA*2–6	1	3	2	7	2	19	N/A		N/A	
GodA*1–3-A1C/T2A	1	3	2	7	2	20	N/A		N/A	
GodA*1–3-A1T/T2A	1	3	2	7	2	21	N/A		N/A	

GodA*1–3- A1S/T2A	1	3	2	7	2	22	N/A	N/A
GodA*1–3-T2A/V3C	1	3	2	7	2	23	N/A	N/A
GodA*1–3-T2A/V3T	1	3	2	7	2	24	N/A	N/A
GodA*1–3-T2A/V3S	1	3	2	7	2	25	N/A	N/A
GodA*1–9-A1T/T2A	1	3	2	7	2	26	N/A	N/A
GodA*1–6-A1T/T2A	1	3	2	7	2	27	N/A	N/A
GodA*1–6-T5I/I6T	1	3	2	7	2	28	N/A	N/A
GodA*1–9-T2V/V3T	1	3	2	7	2	29	N/A	N/A
GodA*1–6-T2V/V3T	1	3	2	7	2	30	N/A	N/A
GodA*1–9-L7C/C8L	1	3	2	7	2	31	N/A	N/A
GodA*1–9--1A1	1	3	2	7	2	32	N/A	N/A
GodA*1–9-3A4	1	3	2	7	2	33	N/A	N/A
GodA*1–9-6A7	1	3	2	7	2	34	N/A	N/A
GodA*1–3-T2C	1	3	2	7	2	35	N/A	N/A
GodA*1–3-T2S	1	3	2	7	2	36	N/A	N/A
GodA*1–3-A1E	1	3	2	7	2	37	N/A	N/A
GodA*1–3-A1R	1	3	2	7	2	38	N/A	N/A
GodA*1–3-A1Y	1	3	2	7	2	39	N/A	N/A
GodA*1–3-A1W	1	3	2	7	2	40	N/A	N/A
GodA*1-3-V3R	1	3	2	7	2	41	N/A	N/A
GodA*1–3-V3Y	1	3	2	7	2	42	N/A	N/A
GodA*1–3-V3W	1	3	2	7	2	43	N/A	N/A

GodA*1–3-V3E	1	3	2	7	2	44	N/A		N/A	
GodA*1–16	1	3	2	7	2	45	N/A		N/A	
GodA*1–13	1	3	2	7	2	46	N/A		N/A	
GodA*-T5A	1	3	2	4	2	47	2	6	N/A	
GodA*-S15A	1	3	2	4	2	5	2	48	N/A	
GodA*-S4A	1	3	2	4	2	49	2	6	N/A	
GodA*-S14A	1	3	2	4	2	5	2	50	N/A	
GodA*LP(-25)–(-1)	1	51	2	4	2	5	2	6	N/A	
GodA*LP(-20)–(-1)	1	52	2	53	2	5	2	6	N/A	
GodA*LP(-15)–(-1)	1	54	2	55	2	6	N/A		N/A	
GodA*LP(-10)–(-1)	1	56	2	57	2	6	N/A		N/A	
GodA*LP(-6)–(-1)	1	58	2	59	2	60	N/A		N/A	
GodA*-10SA11	1	3	2	4	2	61	2	62	N/A	
GodA*-tandem	1	3	2	4	2	63	2	64	2	65

Supplementary Table 7. Primers for site-directed mutagenesis of *godA*.

#	Name	Sequence
1	A1S_sense	TCGACCAACGGCGCCTCCACCGTCAGCACC
2	A1S_anti	GGTGCTGACGGTGGAGGCGCCGTTGGTCGA
3	V3S_sense	AACGGCGCCGCCACCAGCAGCACCATCCTG
4	V3S_anti	CAGGATGGTGCTGCTGGTGGCGGCGCCGTT
5	I6S_sense	GCCACCGTCAGCACCAGCCTGTGCAGCGGC
6	I6S_anti	GCCGCTGCACAGGCTGGTGCTGACGGTGGC
7	I6Y_sense	GCCACCGTCAGCACCTACCTGTGCAGCGGC
8	I6Y_anti	GCCGCTGCACAGGTAGGTGCTGACGGTGGC
9	L7S_sense	ACCGTCAGCACCATCTCGTGCAGCGGCGGC
10	L7S_anti	GCCGCCGCTGCACGAGATGGTGCTGACGGT
11	G10S_sense	ACCATCCTGTGCAGCAGCGGCACCCTCAGC
12	G10S_anti	GCTGAGGGTGCCGCTGCTGCACAGGATGGT
13	G11S_sense	ATCCTGTGCAGCGGCAGCACCCTCAGCTCG
14	G11S_anti	CGAGCTGAGGGTGCTGCCGCTGCACAGGAT
15	L13S_sense	TGCAGCGGCGGCACCTCCAGCTCGGCCGGC
16	L13S_anti	GCCGGCCGAGCTGGAGGTGCCGCCGCTGCA
17	A16S_sense	GGCACCCTCAGCTCGTCCGGCTGCGTCTGA
18	A16S_anti	TCAGACGCAGCCGGACGAGCTGAGGGTGCC
19	G17S_sense	ACCCTCAGCTCGGCCAGCTGCGTCTGATCG
20	G17S_anti	CGATCAGACGCAGCTGGCCGAGCTGAGGGT
21	V19S_sense	AGCTCGGCCGGCTGCAGCTGATCGGTCGTC
22	V19S_anti	GACGACCGATCAGCTGCAGCCGGCCGAGCT
23	V19Y_sense	AGCTCGGCCGGCTGCTACTGATCGGTCGTC
24	V19Y_anti	GACGACCGATCAGTAGCAGCCGGCCGAGCT
25	10SA11_sense	ATCCTGTGCAGCGGCAGCGCCGGCACCCTC
26	10SA11_anti	GAGGGTGCCGGCGCTGCCGCTGCACAGGAT

Supplementary References

1 Furihata, K. & Seto, H. Constant time HMBC (CT-HMBC), a new HMBC technique useful for improving separation of cross peaks. *Tetrahedron Letters* **39**, 7337-7340, doi:10.1016/s0040-4039(98)01574-3 (1998).
